# A New Conformal Cooling Design Procedure for Injection Molding Based on Temperature Clusters and Multidimensional Discrete Models

**DOI:** 10.3390/polym12010154

**Published:** 2020-01-07

**Authors:** Abelardo Torres-Alba, Jorge Manuel Mercado-Colmenero, Daniel Diaz-Perete, Cristina Martin-Doñate

**Affiliations:** Department of Engineering Graphics Design and Projects, University of Jaen, Campus Las Lagunillas, s/n. Building A3-210, 23071 Jaen, Spain; ata00001@red.ujaen.es (A.T.-A.); jmercado@ujaen.es (J.M.M.-C.); ddp00006@red.ujaen.es (D.D.-P.)

**Keywords:** conformal cooling, injection molding, additive manufacturing, industrial design, expert algorithms, Computer Aided Design

## Abstract

This paper presents a new method for the automated design of the conformal cooling system for injection molding technology based on a discrete multidimensional model of the plastic part. The algorithm surpasses the current state of the art since it uses as input variables firstly the discrete map of temperatures of the melt plastic flow at the end of the filling phase, and secondly a set of geometrical parameters extracted from the discrete mesh together with technological and functional requirements of cooling in injection molds. In the first phase, the algorithm groups and classifies the discrete temperature of the nodes at the end of the filling phase in geometrical areas called temperature clusters. The topological and rheological information of the clusters along with the geometrical and manufacturing information of the surface mesh remains stored in a multidimensional discrete model of the plastic part. Taking advantage of using genetic evolutionary algorithms and by applying a physical model linked to the cluster specifications the proposed algorithm automatically designs and dimensions all the parameters required for the conformal cooling system. The method presented improves on any conventional cooling system design model since the cooling times obtained are analogous to the cooling times of analytical models, including boundary conditions and ideal solutions not exceeding 5% of relative error in the cases analyzed. The final quality of the plastic parts after the cooling phase meets the minimum criteria and requirements established by the injection industry. As an additional advantage the proposed algorithm allows the validation and dimensioning of the injection mold cooling system automatically, without requiring experienced mold designers with extensive skills in manual computing.

## 1. Introduction

Injection molding is today the most widely used plastic material processing technology in the industry. The injection molding process is characterized by its high versatility in the manufacture of plastic components by providing parts of complex geometry with good surface finish, high precision and low cost in industrial production [1,2]. Injection molding essentially consists of four stages: injection, packing, cooling, and ejection. During the injection process the plastic material is heated until obtaining a homogeneous melt which is introduced at high pressure into the mold cavity. When the filling of the cavity is almost finished, a subsequent packing pressure is applied which allows the remaining volume of the cavity to be filled, compensating in this way the shrinkage caused by the material cooling. When the material located on the cavity gate solidifies, the process begins of cooling the whole part by thermal exchange with the coolant flow. Once the part is rigid enough to be removed from the mold the part is ejected, and the cycle starts again [3]. In every cyclic manufacturing process cycle time is a key factor in the productivity of the process. In the injection molding process, the time of each cycle depends significantly on the time spent on cooling the molded part [4], this being around 70–80 percent of the cycle time [5,6,7]. Additionally, the quality of the molded part depends to a great extent on the characteristics of the mold cooling process [8,9].

An efficient cooling system plays a vital role in the technology of injection molding, since it optimizes the process of heat exchange between the molded part and the coolant flow. The requirements of the mold cooling process depend fundamentally on the topology of the plastic part, the design and dimensioning of the cooling channels and on the thermal properties of the mold material. In this line, the cooling of the molded part must be as uniform and balanced as possible, so that it makes it possible to eliminate defects such as sink marks, differential shrinkage, residual stress, warping, etc. [10,11]. The design of the cooling channels plays a critical role in the performance of the injection mold [12]. Traditionally, straight cooling channels prevent uniform and effective cooling in free form parts. With the advancement of the additive manufacturing technology the conformal cooling channels mean that the coolant flow can follow the geometry of the molded part, transferring in this way the heat from the cavity to the cooling channel efficiently. Another additional advantage of the conformal cooling channels is the reduction in the cooling time as well as a distribution improvement in the surface temperature of the part. Efficiency in the cooling process is then achieved by designing an optimal channel system that maximizes the process of thermal exchange between the part and the coolant flow. On the other hand, the temperature distribution at the end of the filling phase depends fundamentally on the geometrical features of the part [13]. A complex and non-optimized part topology can cause shearing in the injected material and a lack of uniformity in the surface temperatures. However, despite the importance of prior knowledge of the temperature distribution at the end of the injection phase no method of designing and dimensioning of the cooling system takes this information as input to obtain an optimal and accurate cooling system. Although some researchers focused on the optimization of conformal cooling channel design, there still is no a concrete approach for designing the best cooling system for the mold [14]. Additionally, the number of studies focused on obtaining the design parameters for conformal channels for free form parts automatically is very limited.

To solve these problems, this paper presents a new algorithm for obtaining the automated design of conformal cooling channels. The algorithm surpasses the current state of the art since it uses as input variables firstly the discrete map of temperatures of the melting front in each node of the surface mesh, and secondly a set of geometrical parameters of the part together with the technological and functional requirements of cooling in injection molds. Thus, the dimensioning of the main technological and geometrical parameters of the conformal cooling system is performed via an optimal and precise procedure. In addition, the algorithm allows the grouping, using a discrete format, of geometrical areas of the plastic part in clusters of temperature, from the nodal temperature of the melting front at the end of the filling phase. Finally, the temperature of each cluster coincides with the average temperature of the cluster nodes, which minimizes the standard deviation with respect to the rest of the cluster marginal temperatures. The technological and geometrical parameters obtained after the generation of the clusters is stored in a multidimensional discrete model of the plastic part. With this information it is possible to automatically dimension, by using expert algorithms of the genetic type, all of the parameters required for the cooling system, such as the diameter of the conformal channels and the optimum distance and positioning of each channel with respect to the mold cavity surface. The new algorithm uses the advantages of the process of additive manufacturing in rapid tooling to improve the cooling process in deep zones of the part, eliminating in this way quality failures and achieving as a result a complete and uniform cooling of the plastic part. The algorithm presented here allows the validation and dimensioning of the cooling system of the injection mold without requiring the help of mold makers and expert designers.

## 2. Background and Related Work

The advanced design of conformal cooling channels attracted the attention of designers and mold makers who centered their research on the conformal cooling channels’ design and layout.

An advanced design of the cooling structure for an injection mold can improve the quality of the products and reduce the cycle time. Several studies highlight the fact that a cooling design using conformal cooling channels presents a better performance than a traditional layout with straight channels for cooling free form surfaces [15]. In a first study, Sachs et al. [16] used additive manufacturing with stainless steel powder and a polymer binder to obtain the cavity inserts, integrating a conformal channel lay out into a cylindrical part. In his research he contrasted the performance of the conformal system versus a conventional refrigeration made of steel by means of Computer Numerical Control technologies. The results show a more balanced temperature arrangement in the conformal approach. Nevertheless, the thermal features of the mold material vary using conventional steel, this being an important factor not quantified in the performance of the cooling system. Conforming to these results, Xu et al. [17] developed a new model for sizing the main parameters of the cooling system. In the research presented, Xu divided the mold surface into micro areas called cooling areas in order to obtain the global cooling system. In a second step, the cooling areas were decomposed into cooling units in a space of six design boundary rules for each unit. Finally, the results for each discrete cooling area were again regrouped in order to obtain the cooling system. The results obtained by Xu indicated a reduction of 15% in cycle time and 9% in part warping. Dimla et al. [18] described a procedure for modeling and improving the cooling and heating for injection molds by means of simulated models. Their methodology included a numerical simulation of the designed conformal cooling in order to find the best position for the conformal channels. Quiao et al. [19] applied a procedure for improving the Computer Aided Design model of the conformal channels, analyzing the cooling by means of a sensitivity study established on the boundary elements method and a hybrid optimizer. The sensitivity analysis together with boundary element method presented a good performance; however, they are not applicable to real industrial parts. Kitayama et al. [20] examined the cooling design of conformal channels, analyzing the cycle time and the warpage in industrial molded parts through numerical and experimental tests. The results confirmed that conformal cooling is effective due to the short cycle time and the warpage reduction. Kitayama et al. [21] performed a multi-objective design optimization in order to maximize the minimum weld line temperature for weld lines reduction and to minimize the clamping force, achieving as a result an increase in the quality of the product. Park et al. [22] presented the development of an injection mold with conformal cooling channels for manufacturing a free form industrial component with variable thickness. Their study indicated that conformal cooling channels decrease the cycle time by about 30% in comparison to traditional cooling systems. Park et al. [13] performed a method for maximizing the efficiency of conformal cooling channels manufactured with 3D printing technology. The research results showed that the cooling and cycle time can be reduced by more than 50%. Marques et al. [23] investigated two designs of conformal cooling: the parallel circuit and the serial circuit. Both proposals were evaluated by Computer Aided Engineering simulation to produce parts of polypropylene against traditional cooling circuits (linear channels). The results showed that the deformation of the product can be reduced significantly by using conformal cooling channels. Zink et al. [24] analyzed the effect of mold designing and mold material on cooling efficiency using numerical methods. The simulation model was adjusted to the measurement results by considering the gap between the mold inserts. More recently Jahan et al. [25] proposed a modeling procedure for designing optimized layouts of conformal cooling systems in injection molding. They applied the design of experiments (DOEs) methodology in order to analyze the influence of the main design dimensions, as well as the cross-section topologies. Berger et al. [26] studied the performance of three cooling layouts for cooling a complex-shaped injection molded PA6 GF35 part that exhibited a mass accumulation. The results of the experiments indicated that the 3D printed insert core could greatly improve the uniformity of the surface temperature in the area with defects and reduce the weld line of the manufactured product. Conformal cooling designs were also applied in molds with fast cooling cycles. This technique consists of heating the cavity during filling and cooling the rest of the cycle rapidly. Recent studies can be found in [27,28,29]. Cho et al. [29] studied a 3D printed insert core of an injection molding tool for reducing weld line defects on automotive crash pads with a high standard of appearance. The heat transfer experiments were conducted in order to compare the efficiency of two different types of building up methods for insert cores, and a heat transfer simulation was conducted to design a heating and cooling channel for the conformal surface temperature of the insert core and for thermal response efficiency. For conformal cooling uses in small scale molds Clemente et al. [30] introduced the flow channels svelteness as a total geometric dimensioning value for obtaining their layout.

Unfortunately, all the designs presented had the disadvantage of a totally manual execution. To resolve this issue, several researchers studied the possibility of automating the conformal cooling design for injection molding. To decrease the progressive rise in the coolant temperature from the start point to the final point of the channels lay out, Au et al. [31] designed a methodology to adapt the positioning of the conformal cooling channels to the mold cavity. Wang et al. [32] performed a new procedure for automated designing of the conformal cooling system. Unfortunately, this new procedure required a large quantity of information with regard to the coolant, cycle time, sizing of the cooling channels etc. The values were obtained by the user, and so were partially dependent on designers. Additionally, the cooling channel design developed by Wang was made up of complex networks. To resolve this issue, Wang et al. [33] presented a new methodology for obtaining conformal cooling designs by means of spiral channels. This arrangement improved the cooling channels and decreased production costs. Recently, Li et al. [14] proposed a topology optimization method for designing the conformal cooling layout for injection molds. In the course of the modeling process the cycle-averaged procedure was employed to facilitate the study of the cooling performance, and the boundary element method (BEM) was employed to resolve the governing algorithms. Unfortunately, the channel arrangement required the performance of a self-complied code to obtain the sensitivities.

Additive manufacturing can create three-dimensional lattices and scaffoldings with specific mechanical and thermal properties [34,35,36,37,38]. Several authors developed new cooling proposals using scaffoldings or porous structures. Porous structures can achieve a larger surface-area-to-volume ratio, which may further reduce the thermal inertia of an injection mold. Compared with conventional circular channels, porous structures exhibit better mechanical performance during the injection molding process. Some authors presented new proposals using uniform porous scaffolds with a cubic shape which are used to fill the cooling area uniformly. Au et al. [39] presented a balanced accuracy lattice construction for cooling channels design. The conclusions indicated that this arrangement supplied a more balanced cooling geometry in comparison to traditional cooling layouts and enhanced the mechanical strength of the cavities in the injection mold. In Au et al. [40] they followed up their work by performing a different design procedure comprising of a conformal cooling passageway with porous multi-connected features, established on the theory of duality. The conclusions of their research presented a more balanced mold cooling distribution in comparison with available conformal layouts. Crema et al. [41] presented a new mold insert designed using scaffoldings and validated by numerical simulations for rapid heating. Several other cell topologies, including cross and N-shape cells, were also investigated. Along these lines Brooks et al. [42] presented a study based on using conformal cooling layers designed using unit cells without supports. The results presented a decrease in cooling time of 26% compared to the traditional cooling systems. Unfortunately, there are still some drawbacks to using uniform porous structures to achieve conformal cooling. Firstly, the pressure drop in uniform porous structure is usually much higher than that of conventional cooling channels. Secondly, the distance between the cooling surface and the mold is not constant. To resolve these issues, Tang et al. [43] proposed a general design method of conformal porous structure for injection mold cooling that can achieve better cooling performance by reducing the maximum average mold temperature and the pressure drop. More recently, Mercado et al. [44] proposed a new layout for a conformal cooling system with the surface of a parametric lattice. The parameters of the cooling channels, such as the diameter of the incoming lattice channels, are automatically sized requiring as input only information of the material and the part topology. Even so, the topologies of the lattice arrangements [39,40,41,42,43,44] could originate too much turbulence and pressure drops in the coolant flow as it advances through the scaffolds.

Despite all the advantages of conformal cooling channels, their benefits are maximized only when an automated design method capable of optimizing the heat exchange process between the part and the coolant flow is carried out. The knowledge of the temperature at each point on the part surface at the beginning of the cooling phase helps to increase the accuracy in the dimensioning and arrangement of the channels with respect to the part surface. However, none of the methods presented in the state of the art include as an input variable the non-uniform distribution of temperatures on the surface of the part at the end of the filling phase. Considering a constant injection temperature in the whole part as input for the layout and sizing of the mold cooling can cause important design errors. At the end of the injection phase rheological problems such as an increase in the temperature of some areas of the part due to the shearing of the material, lack of surface thermal uniformity or hot spots caused by a thickness variability can occur. All these factors have a great influence on the geometrical design and sizing of the cooling system. Additionally, for designing cooling channels in injection molding it is essential to include geometrical, functional, and technological requirements that allow us to achieve, at the end of the cooling phase, a uniformity in the part surface temperatures eliminating warpage and residual stresses as well as reducing the cooling time.

To solve these problems this paper presents a new design system for the automated design of conformal cooling channels. The methodology for sizing the geometrical and technological variables of the conformal cooling system is applied, taking into account the geometric discretization of the plastic surface in temperature clusters and the advantages of using expert optimization algorithms of an evolutionary type. In this way the average temperature of the molten plastic front is used as an input parameter to the physical model for the dimensioning of the conformal cooling system, linking the physical model to the requirements of each cluster. It is thereby possible to obtain the magnitude of the geometrical and technological variables of the conformal cooling system associated with each cluster, obtaining as a result an optimum uniformity in the cooling of the molded part. The new proposal employs the benefits of the technology of additive manufacturing (rapid tooling), enhancing the cooling of the deep areas of the geometry and removing quality failures. The new system permits the validation and sizing of the cooling mold design without requiring skills in manual computing and experience on the part of the mold designer.

## 3. Materials and Methods

### 3.1. Discrete Model of the Plastic Part

**Figure 1:**
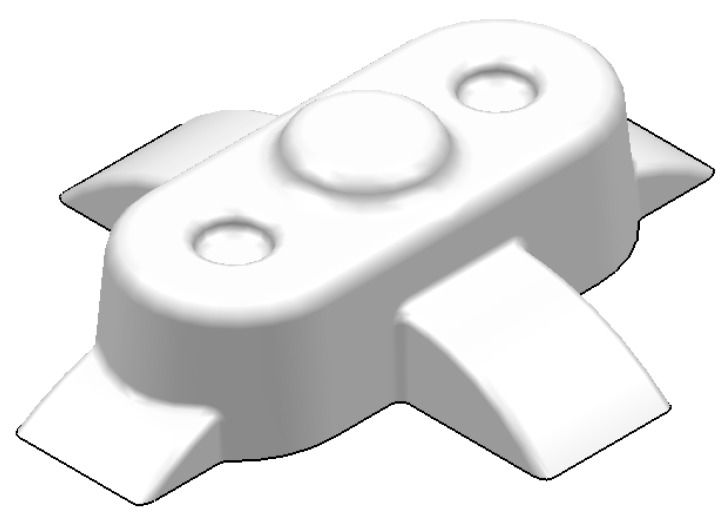
Plastic part virtual model *P*.

**Figure 2:**
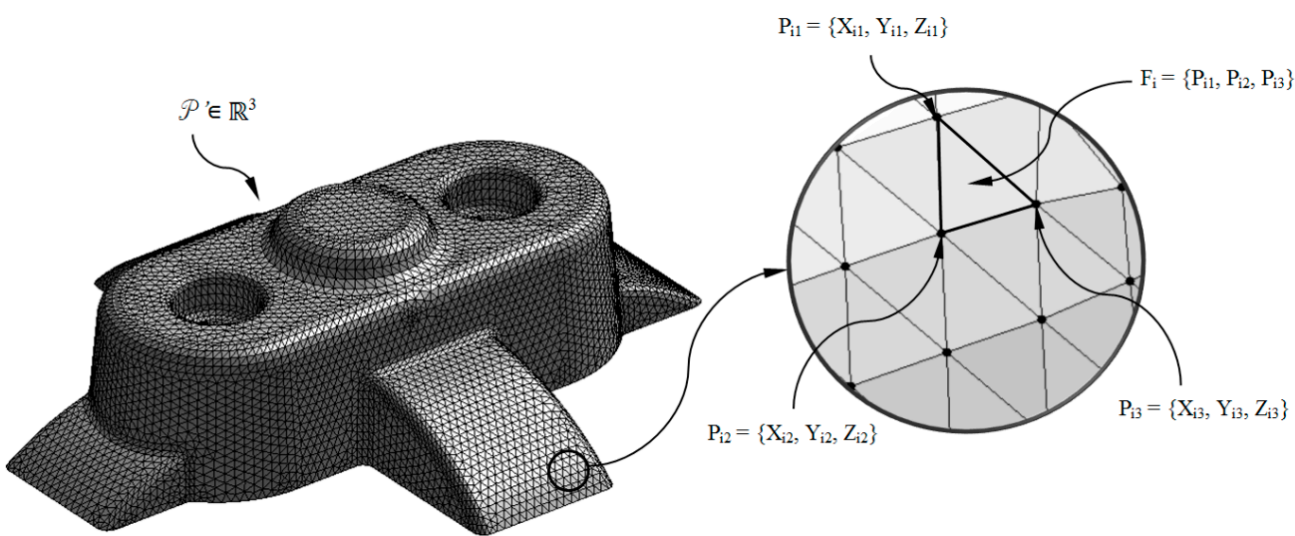
Discrete geometry *P’* ∈ ℝ^3^ of the plastic part and detail of a triangular facet F_i_ and the nodes P_i1_, P_i2_, P_i3._

The algorithm proposed in this paper is based on the previous geometrical analysis of the plastic part under study P ∈ ℝ^3^ (see Figure 1). Throughout this process the 3D geometry of the plastic part is discretized with the aim of obtaining a three-dimensional mesh P’ ∈ ℝ^3^ (see Figure 2). The three-dimensional discrete mesh of the plastic part P’ ∈ ℝ^3^ (see Figure 2) is composed of remarkable elements of the node type P_ij_ = {X_ij_, Y_ij_, Z_ij_} (with the geometrical information of their Cartesian coordinates, see Figure 2) and facets or polygonal surfaces F_i_ = {P_i1_, P_i2_, P_i3_} ∈ P’_f_ (with the geometrical information about the nodes that compose it, see Figure 2). For the methodology proposed in this paper the facets or polygonal surfaces are defined as triangular. Nevertheless, the scope of the proposed methodology can be extended to facets with different geometrical definitions. The accuracy of the mesh or average length of the facets of each of the plastic parts analyzed is establish according their thickness. Figure 2 shows the graphical representation of the plastic part virtual mesh model and in detail the geometry of the triangular facets and the nodes that define it.

### 3.2. Temperature Map of the Melting Front in the Plastic Part

With the aim of designing and dimensioning a conformal cooling system optimized and adapted to the geometrical and technological variables of the plastic part, the algorithm proposed in this paper establishes as an input variable the temperature of the melting front after the filling phase. From this technological variable it is possible to access the melt plastic flow temperature in each area and, in particular, in each node P_ij_ ∈ P’_n_ that forms the discrete geometry of the plastic part. Hence, the magnitude of the technological and geometrical variables of the conformal cooling system are adjusted to the temperature map of the melt plastic flow of the plastic part by optimizing and adapting the cooling system to each geometrical area, improving the final surface quality, the structural behavior, and the functional requirements. It also manages to reduce the residual stress acquired by the gradient of temperatures between different areas of the plastic part as well as a reduction in the cycle time, improving the productivity of the manufacturing process.

#### 3.2.1. Mold Cavity Filling Stage Modeling

**Figure 3:**
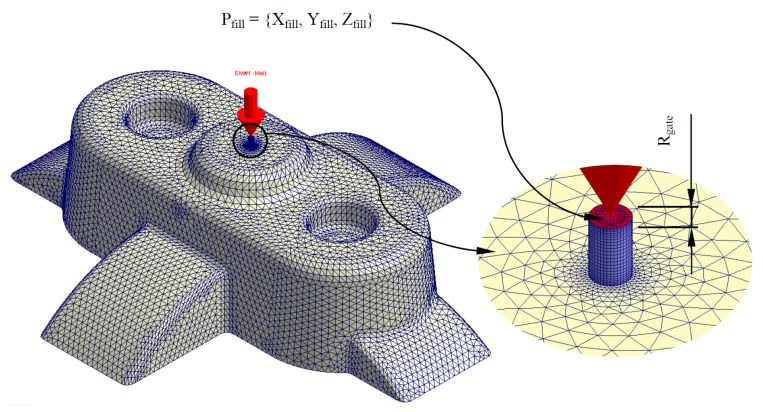
Discrete geometry *P’* ∈ ℝ^3^ of the plastic part and detail of the filling injection point and injection mold gate.

Given the plastic part virtual mesh model P’ ∈ ℝ^3^ and the thermoplastic material of which it is composed, the temperature of the thermoplastic filling flow is defined by a numerical simulation of the filling process in the injection mold cavity. This numerical analysis was carried out using the numerical simulation software Moldex 3D R16 for injection molding [45]. Firstly, the plastic part virtual mesh model under study is imported into the numerical simulation software. For this task the discrete mesh P’ ∈ ℝ^3^ must be previously defined, together with the Cartesian coordinates P_fill_ = {X_fill_, Y_fill_, Z_fill_} ∈ ℝ^3^ and geometrical dimensions of the injection point or gate R_gate_ (see Figure 3).

**Figure 4:**
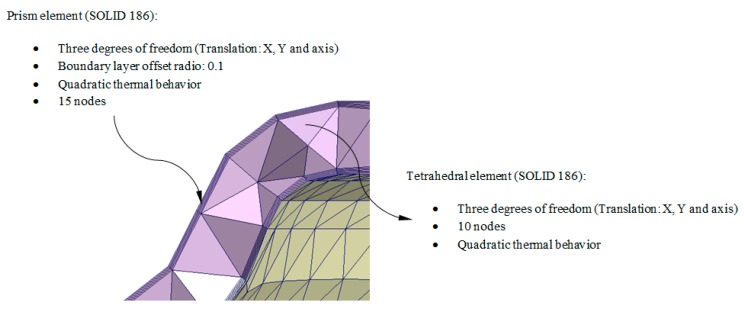
Detail of the three-dimensional elements and boundary layers that make up the mesh for the Computer Aided Engineering simulation of the filling of the geometry under study P.

As shown in Figure 3 and Figure 4, the numerical simulation software for injection molds Moldex 3D R16 discretizes the internal volume of the plastic part from the discrete mesh P’ ∈ ℝ^3^ using SOLID 186 tetrahedron-type elements with average length equal to the precision ε of P’ ∈ ℝ^3^. These elements have a quadratic thermal behavior, being composed of 10 nodes (four nodes on the tetrahedron vertex and six nodes on the midpoints of the edges). The tetrahedrical elements include three degrees of freedom per node, these three nodal translations being in the X, Y and Z directions. Furthermore, to improve the numerical modeling of the contact area between the injection mold surface and the melting front a set of elements with a variable and adaptable size called boundary layers was defined (see Figure 4). This set of elements covers the whole contact surface between the injection mold and the plastic part. Each element is composed of five layers of SOLID 186 prismatic elements whose layer height is established by an offset ratio, which relates the height of the prismatic element and the average size of the element. The offset ratio used for each of the plastic parts analyzed in this paper was equal to 0.100. This value corresponds to the height of the prismatic element in the boundary layers, being equal to the average length of the prismatic element multiplied by the offset ratio. These elements have a quadratic thermal behavior, being composed of 15 nodes (6 nodes in the vertex of the prism and nine nodes in the midpoints) and three degrees of freedom in each node: translation nodal in the X, Y and Z directions. Figure 4 shows the precision, the number of nodes and elements and element type of each of the plastic parts meshes analyzed in this paper.

Next, the thermoplastic material of the plastic part under study is established. The numerical simulation software Moldex 3D R16 has a wide database of thermoplastic materials from different manufacturers, so the material selection is performed during the numerical simulation configuration of the injection mold cavity filling. The thermoplastic materials used for the case studies analyzed in this paper are: Cycolac MG47 (ABS), Finalloy EBP-830 (PP) and Lexan HF1110R (PC). Finally, the technological parameters that govern the numerical analysis of the filling phase are shown in Table 1. The whole set of input technological parameters corresponding to the Cartesian coordinates of the injection points P_fill_ = {X_fill_, Y_fill_, Z_fill_} ∈ ℝ^3^ are determined according to [4], in order to automate the process of numerical simulation during the mold cavity filling. The input technological parameters required for the algorithm were the dimensions of the gate R_gate_, the filling time of the mold cavity T_fill_, the packing time T_pack_ and the cooling time T_cooling_. The remaining technological parameters: melt temperature T_melt_, mold temperature T_mold_, plastic part ejection temperature T_eject_ and maximum injection pressure P_inj_ are automatically defined with the selection of the thermoplastic material, as the manufacturer recommends their magnitude for an optimal cavity filling process of the injection mold. Each of the variables is presented in Table 1, Table 2 and Table 3.

**Figure 5:**
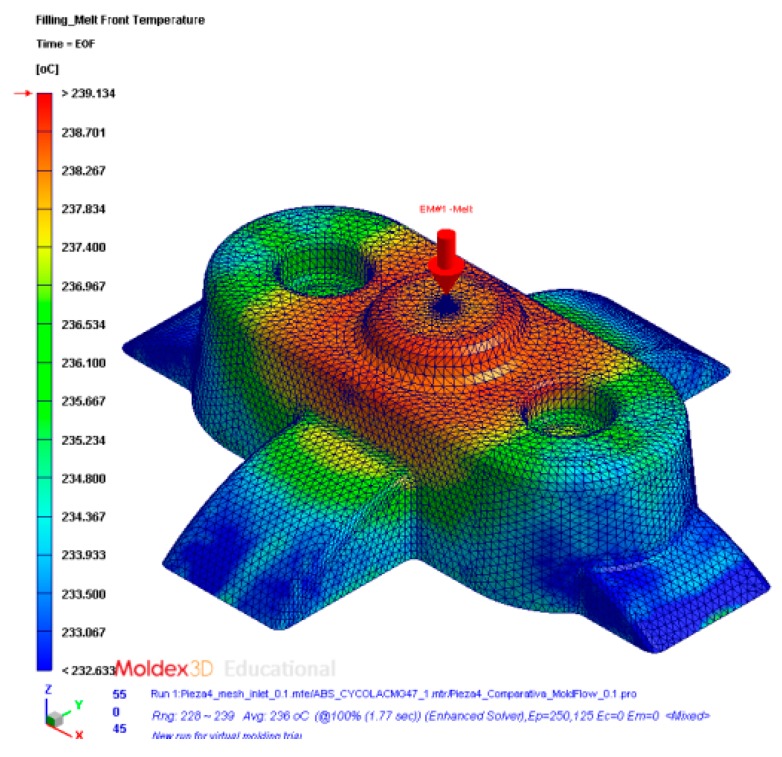
Temperature definition of the melting front during the filling phase of the plastic part.

**Figure 6:**
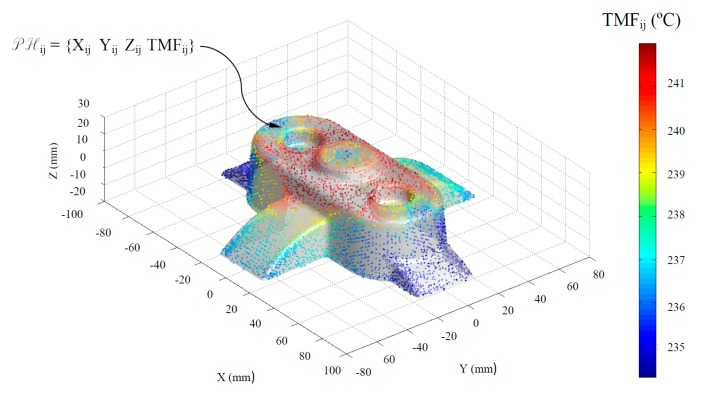
Definition of the hybrid matrix PH*_ij_* from the discrete geometry P’*_n_* ∈ ℝ^3^ and from the temperature of the melt plastic front TMF_ij_ in each node.

Figure 5 and Figure 6 graphically show the temperature map distribution of the melt plastic flow TMF_ij_ ∈ ℝ (°C) (see Equation (1)) in each node of the plastic part virtual mesh model under study. The result of the temperature of the thermoplastic filling flow obtained for each node P_ij_ ∈ P’_n_ belonging to P’ ∈ ℝ^3^ is exported in ASCII format for a later use as parameter for the calculation of the geometrical and technological variables of the conformal cooling system in the proposed algorithm. In this way, by combining the result of the technological variable TMF_ij_ ∈ ℝ obtained from the numerical simulation and the Cartesian coordinates of each node P_ij_ = {X_ij_, Y_ij_, Z_ij_} ∈ P’_n_, it is possible to establish a hybrid matrix PH_ij_ ∈ ℝ^4^ (see Figure 6) of range 4·n x m that combines geometrical information of the part with technological information about its manufacture, conforming a multidimensional discrete model of the plastic part. (see Equation (1)).

(1) $$\forall \;P_{\mathrm{ij}}\;=\;\left\{\begin{array}{ccc} X_{\mathrm{ij}} & Y_{\mathrm{ij}} & Z_{\mathrm{ij}} \end{array}\right\}\in {\;P\;{}^{\prime }}_{n}\;\exists {\;\mathrm{TMF}}_{\mathrm{ij}}\in \mathbb{R}\;|{\;\mathrm{PH}}_{\mathrm{ij}}=\left\{\begin{array}{cccc} X_{\mathrm{ij}} & Y_{\mathrm{ij}} & Z_{\mathrm{ij}} & {\mathrm{TMF}}_{\mathrm{ij}} \end{array}\right\}\in {\mathbb{R}}^{4}$$

#### 3.2.2. Clustering of Discrete Geometrical Areas Via Melting Front Temperature

**Figure 7:**
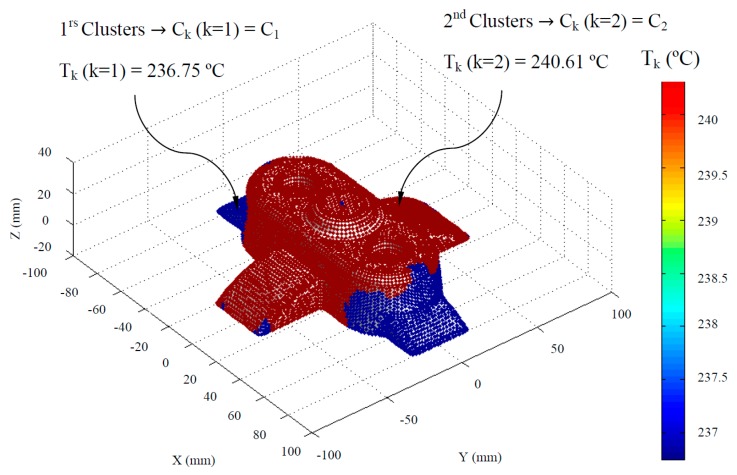
Definition of the geometrical discretizations or clusters of the plastic part.

The temperature definition of the thermoplastic filling flow establishes the temperature TMF_ij_ ∈ ℝ, in each node P_ij_ ∈ P’_n_ during the filling phase. To dimension in an optimized manner, the main technological and geometrical variables of the channels that conform to the cooling system, the nodes P_ij_ ∈ P’_n_ of the discrete mesh P’ ∈ ℝ^3^ are grouped based on the temperature of the thermoplastic filling flow TMF_ij_ ∈ ℝ. Hence, it is feasible to dimension the elements of the cooling system adapted to the technological requirements of the plastic part to be molded. Thus, it is possible to perform discrete groupings of geometrical areas of the plastic part C_k_ (see Figure 7 and Equation (2)) whose nodal temperature of the melting front is similar.

(2) $$\forall \;{\;P\;{}^{\prime }}_{n}\in {\mathbb{R}}^{3}\;\exists \;C_{k}\;|\;{\mathrm{PH}}_{\mathrm{ij}}=\left\{\begin{array}{cccccc} X_{\mathrm{ij}} & Y_{\mathrm{ij}} & Z_{\mathrm{ij}} & {\mathrm{TMF}}_{\mathrm{ij}} & C_{k} & T_{k} \end{array}\right\}\in {\mathbb{R}}^{6}\;\forall k\in \left\{\begin{array}{ccc} 1 & \ldots & n_{\mathrm{clusters}} \end{array}\right\}$$

Where n_clusters_ represents the number of discrete groups of geometrical regions of the plastic part, C_k_ the index of the discrete groups or clusters and T_k_ (°C) the average temperature of the melt plastic flow for each discrete group or cluster. In this way the range of the hybrid matrix PH_ij_ ∈ ℝ^6^ becomes 6·n x m, expanding the information of the discrete multimodal model to include technological and geometrical parameters obtained after the generation of the clusters. Therefore, it is possible to establish from the hybrid matrix PH_ij_ ∈ ℝ^6^ the index C_k_ and the average temperature of the molten plastic front T_k_ of the discrete clusters to which each node P_ij_ ∈ P’_n_ belongs.

The discrete groups of geometrical areas of the plastic part are established by means of the statistical algorithm of generation of k-means clusters [46,47]. This algorithm has as a main objective the division of a set of n observations into a determined number of k subsets or clusters. So each observation belongs to the subset or clusters whose average value is closer. For the application of the k-means algorithm to the methodology proposed in this paper the temperature of the melt plastic flow TMF_ij_ ∈ ℝ is established as the initial set of observations, which in turn are related to the Cartesian coordinates of each node P_ij_ ∈ P’_n_ by the hybrid matrix PH_ij_ ∈ ℝ^6^.The temperature of the melt plastic flow TMF_ij_ ∈ ℝ is a vector (vector column of the hybrid matrix PH_ij_ ∈ ℝ^6^), and from it the subsets or clusters C_k_ are constructed in order to minimize the sum of the squares of the average temperatures of the melt plastic flow in each of them (see Equation (3)).

(3) $$\begin{array}{c} \arg\min\\ C_{k} \end{array}\overset{n_{\mathrm{clusters}}}{\underset{k=1}{\sum }}\underset{{\mathrm{TMF}}_{\mathrm{ij}}\in C_{k}}{\sum }\|{\mathrm{TMF}}_{\mathrm{ij}}-T_{k}{\|}^{2}\;\forall k\in \left\{\begin{array}{ccc} 1 & \ldots & n_{\mathrm{clusters}} \end{array}\right\}$$

To establish an optimal number of discrete groups of geometrical areas of the plastic part or clusters, the Davies-Bouldin clustering evaluation criterion [48] is included. The Davies-Bouldin criterion is based on a ratio of within-cluster and between-cluster distances. The Davies-Bouldin index DB is defined as shown by Equation (4).

(4) $$\mathrm{DB}=\frac{1}{n_{\mathrm{clusters}}}\cdot \overset{n_{\mathrm{clusters}}}{\underset{u=1}{\sum }}\max_{u\neq v}\left\{D_{u,v}\right\}$$

where D_u,v_ is the within-to-between cluster distance ratio for the u and v clusters. Equation (5) shows this parameter in mathematical terms.

(5) $$D_{u,v}=\frac{{\bar{d}}_{u}+{\bar{d}}_{v}}{d_{u,v}}$$

where ${\bar{d}}_{u}$ is the average distance between each point in the u cluster and the centroid of the u cluster, ${\bar{d}}_{v}$ is the average distance between each point in the v cluster and the centroid of the v cluster, and d_u,v_ is the Euclidean distance between the centroids of the u and v clusters. The maximum value of D_u,v_ represents the worst-case within-to-between cluster ratio for cluster u. The optimal clustering solution has the smallest Davies–Bouldin index value. In a similar manner, in order to improve the convergence criterion of the algorithm a vector V_k_ ∈ ℝ was proposed, from which the optimum number of discrete groups of geometrical areas of the plastic part or clusters will be established (see Equation (6)).

(6) $$V_{k}=\left\{\begin{array}{ccc} n_{{\mathrm{clusters}}_{\min}} & \ldots & n_{{\mathrm{clusters}}_{\max}} \end{array}\right\}\in \mathbb{R}$$

where n_clusters min_ represents the minimum number of optimal clusters and n_clusters max_ represents the maximum number of optimal clusters. For the algorithm established in this manuscript the magnitude of these parameters is 2 and 6, respectively.

### 3.3. Dimensioning of the Conformal Cooling Channels via Genetic Algorithms

The calculation process of the technological and geometrical variables magnitude of conformal cooling channels via genetic optimization algorithms is described in the present section. The use of this type of algorithms allows that the geometrical design of the conformal cooling system to be adapted to a set of boundary conditions of cooling in injection molds. In this way it is possible to optimize in turn the heat exchange process between the coolant flow and the injection mold cavity.

Table 2 describes the technological and geometrical variables of the conformal cooling channels that the genetic algorithm proceeds to optimize. It should be noted that this dimensioning process is carried out according to the physical model presented and, in turn, it is applied taking into account each of the geometrical discretizations or clusters (see Figure 7) previously defined, i.e., since each geometrical discretization or cluster C_k_ has an associated average temperature of the thermoplastic filling flow T_k_, the present dimensioning process is adjusted to the technological variables and conditions of each geometrical discretization or cluster C_k_.

The magnitude of the thermal and technological properties of the injection mold material as well as the plastic part thermoplastic material were established from the manufacturer´s information. In addition, it should be noted that the magnitude of the variable temperature of the thermoplastic filling flow T_melt_ (°C) is determined from the magnitude of the average temperature of the thermoplastic filling flow T_k_ (°C), for each geometrical discretization or previously defined C_k_ cluster.

As shown in Table 2, the number of variables of the genetic optimization algorithm for dimensioning the geometrical and technological variables of the cooling channels is not constant. The number of variables is a function of the number of previously defined geometrical clusters (see Figure 7) (minimum 2, maximum 6). In this way, it is established that the variable inlet temperature of the coolant flow T_coolant_ (°C), the separation distance between cooling conformal channels L_conformal_ (m) and the diameter of the conformal cooling channels D_conformal_ (m) are constant for the whole domain of the plastic part regardless of the geometrical cluster discretization to which they belong (see Figure 7). However, the variable of separation distance between the surface of the plastic part and the conformal cooling channels varies depending on the geometrical discretization or clusters (see Figure 7) to which they belong. In this way it is possible to develop a conformal cooling system that allows a uniform temperatures map to be obtained after the cooling phase around the plastic part surface.

Genetic optimization algorithms demand the implementation of a set of fitness functions to be optimized. These functions are related to the boundary conditions defined during the conformal cooling channels design process. Furthermore, constraint limits are defined to meet the additive manufacturing requirements. Table 3 describes the set-up of the genetic optimization algorithm for the theoretical model proposed.

Where T_mold min_ (°C) and T_mold max_ (°C) are defined as suggested values of the thermoplastic material manufacturer, L_min_ (mm), L_max_ (mm), D_max_ (mm) and D_min_ (mm) are the maximum and minimum separation distance between any element of the conformal cooling system (including the plastic part) and the maximum and minimum diameter of the conformal cooling channels, respectively. The magnitude of these geometrical parameters is recommended by the metallic material manufacturer in order to achieve the additive manufacturing criteria without using supports [43]. Table 4 describes the constraint values of the optimization problem variables for each material used in this paper.

To finalize the genetic optimization algorithm definition, the three objective functions that compose it are defined. The first fitness function (see Equation (7)) reduces the sum of differences between the temperature of the mold cavity surface T_mold_ (°C), x(1) (optimization problem variable) and its final magnitude according to the proposed theoretical model (see Appendix A) for each geometrical discretization or cluster of the plastic part (see Figure 7).

(7) $${\mathrm{fitness}\;\mathrm{function}}_{1}:\min\left\{x\left(1\right)-T_{\mathrm{mold}}\right\}\;{\mathrm{Fitness}\;\mathrm{Function}}_{1}:\min\left\{\overset{n_{\mathrm{clusters}}}{\underset{k=1}{\sum }}\left(x\left(1\right)-\left[x\left(2\right)+\frac{\rho_{p}\cdot C_{p}\cdot T_{p}\cdot \left(2\cdot \delta_{s}\cdot x\left(3\right)+h\cdot \pi \cdot x\left(4\right)\cdot x\;(4+k\right)\cdot \left(T_{k}-T_{\mathrm{eject}}\right)}{h\cdot \pi \cdot x\left(4\right)\cdot \delta_{s}\cdot t_{\mathrm{cooling}}}\right]\right)\right\}$$

where k represents the index of each geometrical discretization or clusters and T_k_ (°C) the average temperature of the thermoplastic filling flow for each geometrical discretization or clusters. The second fitness function (see Equation (8)) minimizes the technological variable cooling time t_cooling_ (°C), allowing the production process to be more effective and decreasing costs related to it. Furthermore, residual stress on plastic parts after the cooling phase is an important issue that usually is associated with the final quality of the product. Thus, the established constraints of the technological variables associated with the cooling process temperatures avert this type of defects from happening in the final product.

(8) $${\mathrm{Fitness}\;\mathrm{Function}}_{2}:\mathrm{Min}\left\{t_{\mathrm{cooling}}\right\}=\mathrm{Min}\left\{\frac{T_{h}^{2}}{\pi^{2}\cdot \alpha_{s}}\cdot \mathrm{Ln}\left(\frac{4}{\pi }\cdot \frac{T_{\mathrm{melt}}-x\left(1\right)}{T_{\mathrm{eject}}-x\left(1\right)}\right)\right\}$$

The third fitness function (see Equation (9)) minimizes the sum of the differences between the average temperatures obtained on the surface of the injection mold, according to the proposed physical model (see Appendix A), for each geometrical discretization or clusters of the plastic part (see Figure 7). The definition of this objective function is to obtain a conformal cooling system whose temperature map after the cooling phase and cycle time is uniform for the whole domain of the plastic part.

(9) $${\mathrm{Fitness}\;\mathrm{Function}}_{3}:\min\left\{\overset{n_{\mathrm{clusters}}}{\underset{k=1}{\sum }}\left(\mathrm{diff}\left(x\left(2\right)+\frac{\rho_{p}\cdot C_{p}\cdot T_{p}\cdot \left(2\cdot \delta_{s}\cdot x\left(3\right)+h\cdot \pi \cdot x\left(4\right)\cdot x\;(4+k\right)\cdot \left(T_{k}-T_{\mathrm{eject}}\right)}{h\cdot \pi \cdot x\left(4\right)\cdot \delta_{s}\cdot t_{\mathrm{cooling}}}\right)\right)\right\}$$

Finally, the results achieved by the genetic optimization algorithm in dimensioning the geometrical and technological variables of the conformal cooling channels are analyzed by the numerical simulation software Moldex 3D R16. In this way it is possible to validate the design of the resulting conformal cooling system.

### 3.4. Geometrical Design of the Conformal Cooling Channels Pathing

**Figure 8:**
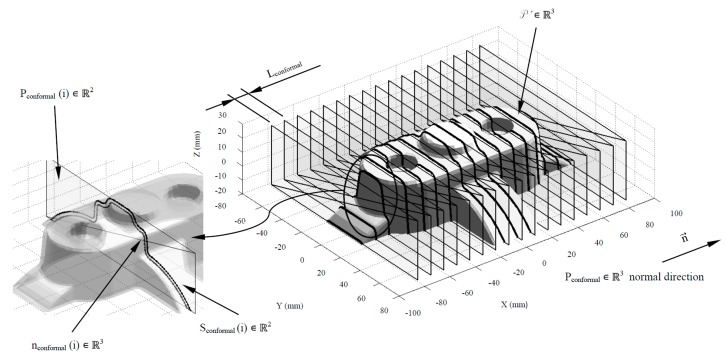
Definition of S_conformal_(i) ∈ ℝ^2^, n_conformal_(i) ∈ ℝ^2^ and P_conformal_(i) ∈ ℝ^3^ for the generation of the layout of the conformal cooling channels.

The algorithm for obtaining the automated geometrical design of the conformal cooling channels takes as a basis the results of the dimensioning expert algorithms and the advantages of an arrangement of channels in zig-zag. The conformal cooling channels design begins with the generation of a set of flat sections S_conformal_ ∈ ℝ^2^ (see Equation (10)) obtained by intersecting the set of planes P_conformal_ ∈ ℝ^3^ (see Figure 8) with the discrete geometry of the plastic part P’ ∈ ℝ^3^ (see Figure 2). The planar sections S_conformal_ ∈ ℝ^2^ are separated from each other by the parametrized distance L_conformal_ obtained from the dimensioning genetic optimization algorithms. Similarly, each planar section S_conformal_(i) ∈ ℝ^2^ is defined by a set of n_conformal_(i) ∈ ℝ^3^ nodes generated by the intersection of the set of P_conformal_ ∈ ℝ^3^ and the edges of the facets that conform to the discrete geometry of the plastic part P’ ∈ ℝ^3^ (see Equation (10)). The normal direction that defines the set of planes P_conformal_ ∈ ℝ^3^ is coincident with the longitudinal direction of the plastic part (see Figure 8) established from the edge of the bounding box that presents a larger dimension related to the layout of the zig-zag conformal cooling channels.

(10) $$\forall {\;P}_{\mathrm{conformal}}\left(i\right)\in {\mathbb{R}}^{2}\;\exists \;\left(\begin{array}{c} S_{\mathrm{conformal}}\left(i\right)\in {\mathbb{R}}^{2}\\ n_{\mathrm{conformal}}\left(i\right)\in {\mathbb{R}}^{2} \end{array}\right)|\left(\begin{array}{c} S_{\mathrm{conformal}}\left(i\right)\in {\mathbb{R}}^{2}\\ n_{\mathrm{conformal}}\left(i\right)\in {\mathbb{R}}^{2} \end{array}\right)=\;P\;{}^{\prime }\cap^{\;}P_{\mathrm{conformal}}\left(i\right)$$

**Figure 9:**
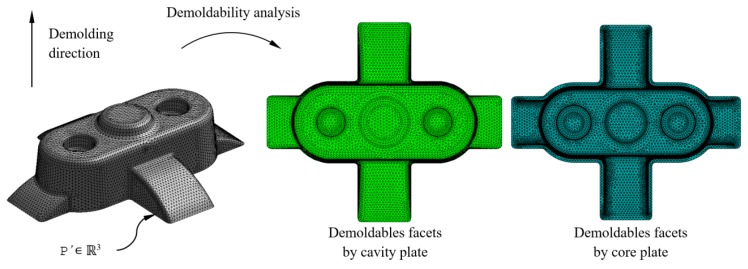
Demoldability analysis of the plastic part discrete geometry.

**Figure 10:**
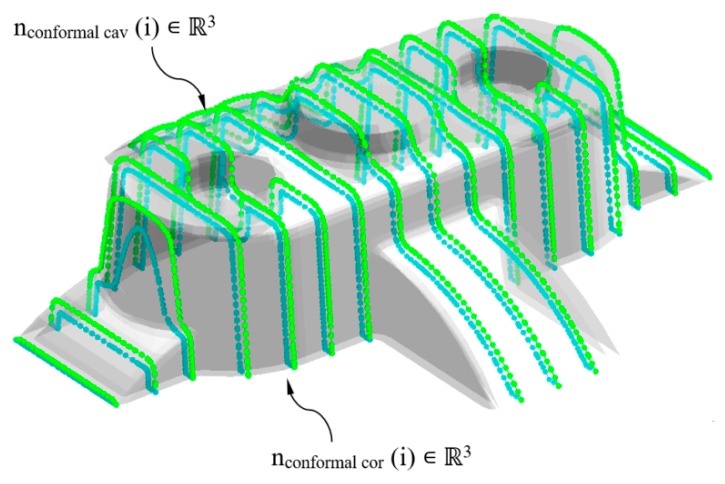
Definition of n_conformal cav_ ∈ ℝ^3^ y n_conformal cor_ ∈ ℝ^3^ for the generation of the pathing of the conformal cooling channels.

The geometrical design of the main plates, cavity, and core, which conforms the injection mold, is defined using the analysis of the plastic part demoldability. The main components of the injection mold cooling system have to be distributed uniformly along the main plates of the mold in order to optimize the cooling process. With the objective of distributing the conformal cooling channel paths in a uniform way along the cavity and core plates, the nodes n_conformal_ ∈ ℝ^3^ belonging to the flat sections S_conformal_ ∈ ℝ^2^ are classified according to their demoldability. The classification of the nodes n_conformal_ ∈ ℝ^3^ is established by evaluating the demoldability of the facets P’_f_ ∈ ℝ^3^ in which the nodes are located. The demoldability of the facets P’_f_ ∈ ℝ^3^ of the discrete geometry P’ ∈ ℝ^3^ is obtained from [1] (see Figure 9). As shown in Figure 10, with this classification two subset of nodes are determined, n_conformal cav_ ∈ ℝ^3^ and n_conformal cor_ ∈ ℝ^3^ (see Equation (11)), each of them defining the geometry of the cooling channel paths housed in the cavity and core plates respectively.

(11) $$\begin{array}{rr} \forall {\;P}_{\mathrm{conformal}}\left(i\right)\in & {\mathbb{R}}^{2}\;\exists {\;n}_{\mathrm{conformal}}\left(i\right)\in {\mathbb{R}}^{2}|{\;n}_{\mathrm{conformal}}\left(i\right)\\ & =\left\{\begin{array}{cc} n_{{\mathrm{conformal}}_{\mathrm{cav}}}\left(i\right) & n_{{\mathrm{conformal}}_{\mathrm{cor}}}\left(i\right) \end{array}\right\} \end{array}$$

**Figure 11:**
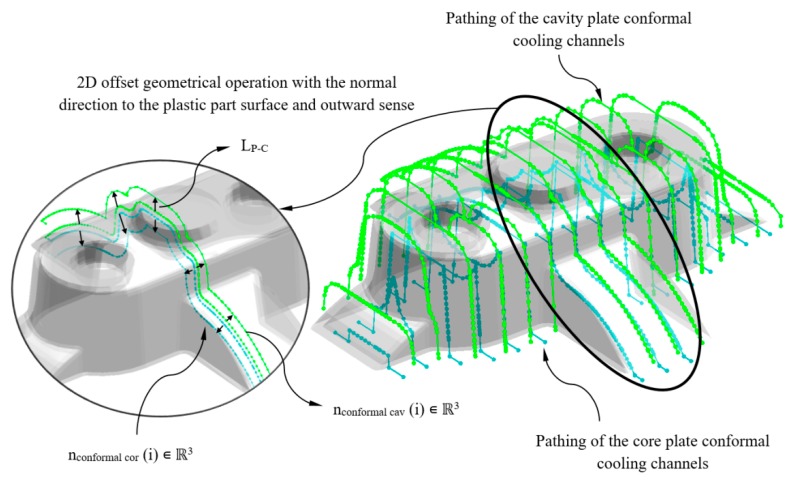
2D offset geometrical operation for defining the conformal cooling channels for the main plates of the cavity and core injection mold.

The set of elements that make up the cooling system of an injection mold are located at a certain distance from the surface of the plastic part. This value is defined on the basis of the geometrical and technological requirements of the plastic part as well as of the injection mold. The methodology presented in this paper focuses on adapting the design of the conformal cooling channels by generating parameterized paths located to variable distances regarding the part surface geometry, taking into account the requirements of the manufacturing process. Therefore, the separation distance between each of the n_conformal cav_ ∈ ℝ^3^ and n_conformal cor_ ∈ ℝ^3^ with respect to the surface of the plastic part is defined by its proximity to the nodes P’_n_ ∈ ℝ^3^ belonging to the hybrid matrix PH_ij_ ∈ ℝ^6^ (see Figure 11). In turn, each cluster C_k_ (see Figure 7) of the hybrid matrix PH_ij_ ∈ ℝ^6^ has an associated separation distance between the cooling channels and the L_P-C_ plastic part. The value of L_P-C_ is defined during the dimensioning process by using genetic optimization algorithms. In this way, once the separation distance between the nodes n_conformal cav_ ∈ ℝ^3^ and n_conformal cor_ ∈ ℝ^3^ and the discrete geometry of the plastic part P’ ∈ ℝ^3^ are determined, a geometrical bidirectional offset is designed following the normal direction to the plastic part surface in an outward sense (see Figure 11).

**Figure 12:**
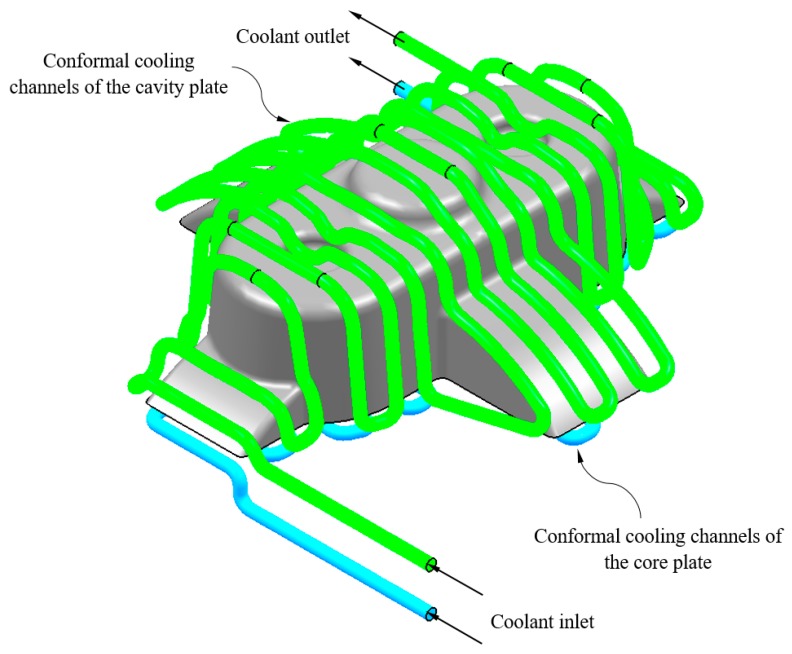
Three-dimensional Computer Aided Design modeling of conformal type cooling channels.

Subsequently, as a result of the 2D offset geometrical operation the nodes n_conformal cav_ ∈ ℝ^3^ and n_conformal cor_ ∈ ℝ^3^ are defined and displaced a distance L_P-C_ with respect to the surface of the plastic part. The nodes n_conformal cav_ ∈ ℝ^3^ y n_conformal cor_ ∈ ℝ^3^ define the axes of the main paths of the cooling channels. To achieve this, a set of smoothed lines are modeled by using rounding radius with a diameter equal of those to the conformal cooling channels D_conformal_. Finally, the algorithm performs a join operation in order to link all the individual lines designing the zig-zag path. The path starts from the polylines that are sited at the end of the plastic part that coincides with the inlet and outlet of the conformal cooling system (see Figure 12). A sweeping algorithm using a circular profile predimensioned by the expert system performs the final layout of the optimal conformal cooling distribution (see Figure 12).

### 3.5. Numerical Analysis of the Conformal Cooling System

**Figure 13:**
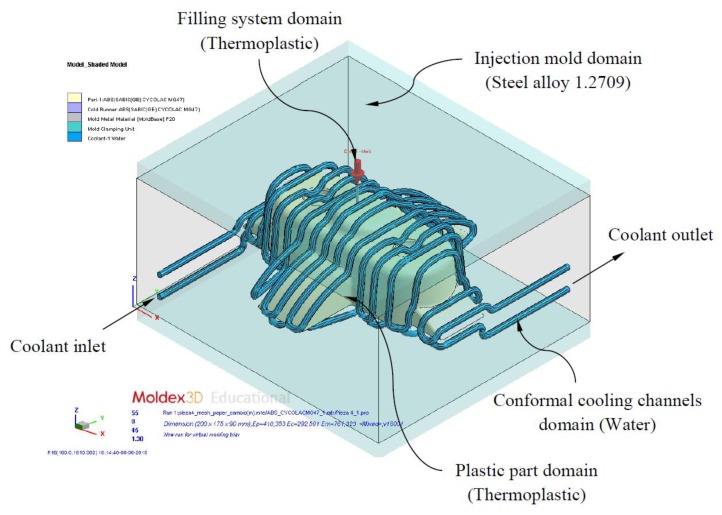
Definition domains and boundary conditions of thermal modeling.

This section describes the configuring process for performing the numerical analysis of the study cases. The main objective of the analysis is to evaluate and validate the thermal performance of the results obtained according to the technological requirements demanded in the injection molding industry. The thermal and rheological numerical simulations analyze the behavior of the coolant flow along the conformal cooling system and the thermal exchange between it and the plastic part during the cooling phase. The set of thermal and rheological simulations are performed in the commercial numerical software Moldex 3D R16 [45]. As shown in Figure 13, the set of solid elements analyzed was divided into three main domains: conformal cooling channels (coolant domain), injection mold (solid domain alloy steel 1.2709), filling system (thermoplastic solid domain) and plastic part (thermoplastic solid domain). The hypotheses defined in the numerical simulations performed are as follows:

- The materials used were: Water (Pure) for the coolant domain, steel alloy 1.2709 for the injection mold domain and the thermoplastics Cycolac MG47 (ABS), Finalloy EBP-830 (PP) and Lexan HF1110R (PC) for the domain of the plastic part and filling system. Table 5 shows the magnitude of the main properties of these materials.
- Since the complete cooling process of the plastic part is analyzed over time, the type of numerical analysis used is “Cooling transient”.
- The total time of the numerical analysis in each simulation covers 30 s, with a magnitude of multiple time steps output equal to 10. For each time step the numerical calculation software stores the solution obtained. In this way, the complete evolution of the temperature map of the plastic part can be evaluated throughout the cooling phase.
- The physical and thermal properties of the coolant flow along the conformal cooling system are analyzed using the Run 3D cooling channels analysis option
- The type of solver used for the numerical analysis of the cooling phase is of the maximum variation of mold temperature type, the temperature difference being equal to 1° and the maximum number of cycles C being 10.
- The turbulence model of the numerical software is activated to analyze the contact of the coolant flow with the walls of the conformal cooling channels. The magnitude of the roughness defined on this contact surface is equal to 0.02 mm

Also, Table 6 shows the configuration parameters of the numerical simulations and the magnitude of the geometrical and technological variables obtained for each case study analyzed in this paper.

As shown in Figure 13, each case study presents boundary conditions of the type inlet and outlet of coolant flow. On the one hand, the coolant flow inlet temperatures are shown in Table 6, and on the other hand the coolant flow inlet velocity is set so that the dimensionless Reynold number is maintained above 4000 throughout the entire system path cooling that is in turbulent regime.

To discretize all the domains shown in Figure 13, three-dimensional elements of the tetrahedron and prismatic type (Solid 186, 10 and 15 nodes, see Figure 4) were used. To define the mesh a dimensioning operation that determines the approximate size of each element of the mesh is used, according to an average length equal to the precision ε of discrete geometry P’ ∈ ℝ^3^ (see Table 7). Furthermore, in order to improve the quality of the mesh in the two interfaces formed by the contact areas between the plastic part and mold surface and the mold surface and the conformal cooling channels, five layers of SOLID 186 prismatic elements were defined (see Figure 4). The offset ratio used in this paper, defined as the ratio between tetrahedron elements size and prismatic elements size is equal to 0.1.

## 4. Implementation and Results

To demonstrate the benefits of the proposed methodology, it was applied to four industrial parts with complex shapes, manufactured by means of plastic injection technology. The accuracy of the nodal mesh (ε) for each geometry is set according to the dimension of the smallest detail of the part. The algorithms were developed in the software Moldex3D R16 [45] (to perform the cooling numerical simulations) and Matlab R2013a [49], Catia V5-6R2016 [50] (to define the geometries of the cooling channels) and with an MSI notebook with an Intel (R) Core (TM) i-77700HQ CPU @ 2.80 GHz. The 3D modeling of the conformal cooling channels was automated by creating an application in the programming environment of the CAD software Catia V5-6R2016 [50].

The proposed methodology establishes the automated design of conformal cooling, based firstly on the topology and geometrical features of the plastic part and secondly on the temperature map during the injection phase, without applying post-processing operations. The location of the cooling elements and their technological parameters are automatically defined. To validate the proposed methodology, several numerical simulations were performed using the injection molding simulation software Moldex3D R16 [45]. The proposed methodology provides a discrete multidimensional model that includes the X, Y and Z coordinates of the axes of the conformal cooling channels, and a set of geometrical and technological variables obtained from the genetic optimization algorithm in order to dimension the cooling system. All this information in discrete format is imported into the software Catia V5-6R2016 [50] to generate the CAD geometry of the cooling system.

Cycolac MG47 (Acrylonitrile butadiene styrene, ABS), Lexan HF1110R (Polycarbonate, PC) and Finalloy EBP-830 (Polypropylene, PP) are the thermoplastic materials used to apply the proposed methodology to the case studies included in this manuscript. On the other hand, structural steel alloy 1.2709 is employed as the material of the plates and components of the injection mold. The materials (Cycolac MG47-ABS, Lexan HF1110R-PC, Finalloy EBP-830-PP and structural steel alloy 1.2709) and geometrical properties used for validating the proposed methodology are indicated in Table 4, Table 5, Table 6 and Table 7. The coolant flow used for the numerical simulations was water with a Reynolds number of 1.5 × 10^4^ (turbulent flow). The inlet temperature of the coolant flow is obtained as a result of the expert algorithm developed for dimensioning the technological variables associated with the injection mold cooling phase.

The final design of the conformal cooling system for the mold that will manufacture the four plastic parts under study is shown graphically in Case A, B, C, D. Table 8 shows the magnitude of the geometrical features required for the analysis of the injection process of the plastic parts. To validate the proposed methodology, the variables resulting from the numerical analyses were coolant temperature, pressure map of conformal cooling channels, surface temperature of the part after the cooling phase and surface temperature gradient of the injection mold. 

The results of the geometrical and technological variables obtained with the genetic optimization algorithm are included for each plastic part analyzed in this manuscript in Table 9, Table 10, Table 11 and Table 12. Table 9, Table 10, Table 11 and Table 12 include the cooling time and the ejection time of the numerical results established for each plastic part analyzed. The cooling time and ejection time value is defined as the time after which the temperature of the mold surface is less than 10 °C. This requirement is established by the industrial sector in the injection molding area as a criterion to guarantee an optimum level of quality in the injected plastic part. As can be seen in Table 9, Table 10, Table 11 and Table 12, the cooling time obtained for the numerical analyses is close to the analytical and ideal cooling time. This value is determined from the geometrical features of the plastic part and the thermoplastic material. The relative error for the plastic geometries analyzed is: 3.118%, 5.000%, 3.225% and 3.776%, respectively.

In this way, it is possible to validate the thermal efficiency of the proposed conformal cooling system based on the results obtained against the ideal cooling solution of the numerical software, resulting in a minimum relative error between both solutions. Conventional cooling systems have large differences with respect to analytical or ideal cooling times, particularly in plastic parts with deep concavities, slender details, internal bosses, etc.

### 4.1. Case A

**Figure 14:**
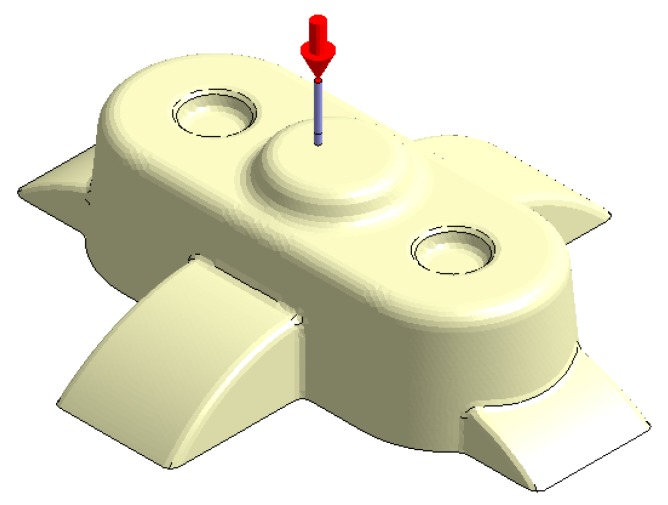
Plastic part, Case A.

**Figure 15:**
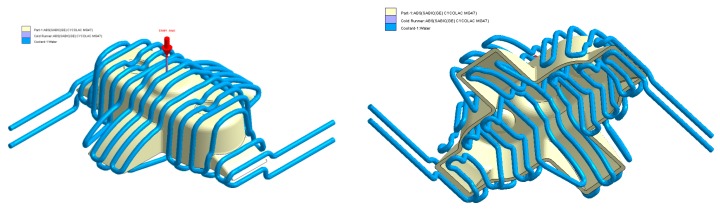
Cooling system design, Case A. Front view (**left**) and bottom view (**right**).

**Figure 16:**
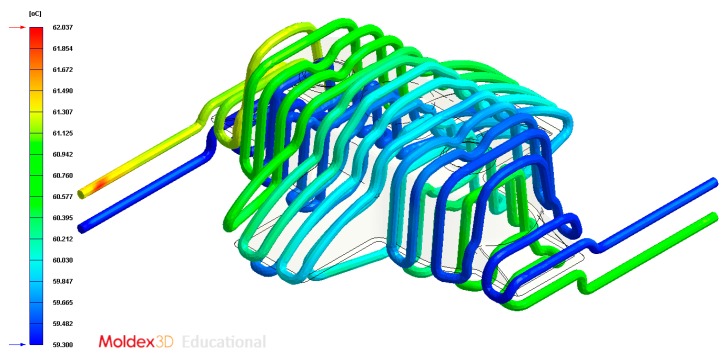
Temperatures (°C) of the coolant along the conformal cooling system, Case A.

**Figure 17:**
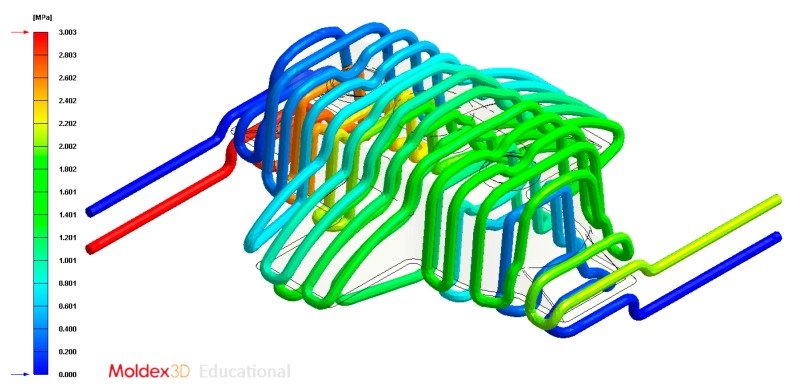
Pressure (MPa) of the coolant along the cooling system, Case A.

**Figure 18:**
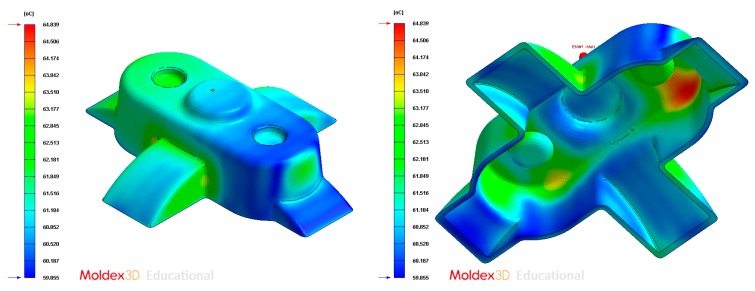
Average temperature (°C) at the surface of the plastic part after the cooling phase, Case A. Front view (**left**) and bottom view (**right**).

**Figure 19:**
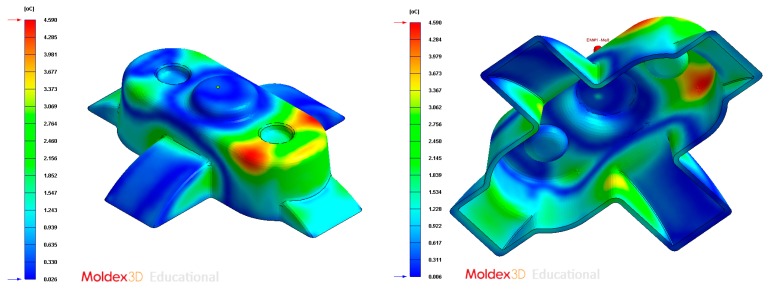
Gradient temperature (°C) on the mold surface after the cooling phase, Case A. Front view (**left**) and bottom view (**right**).

In this section the results obtained for Case A are presented in Figure 14, Figure 15, Figure 16, Figure 17, Figure 18 and Figure 19.

### 4.2. Case B

**Figure 20:**
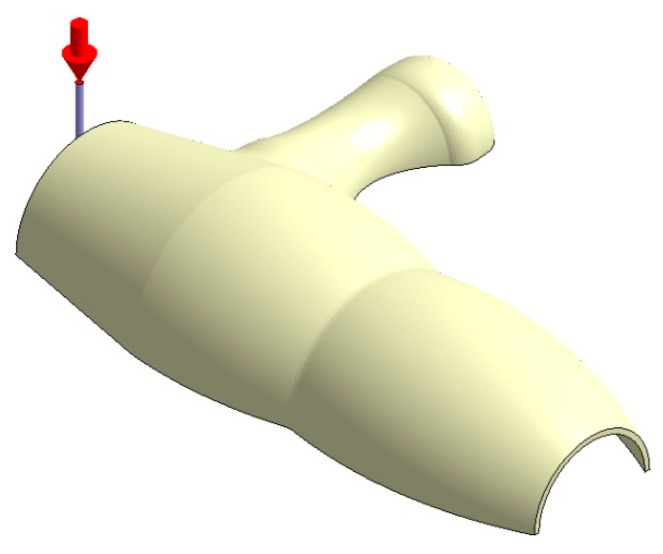
Plastic part, Case B.

**Figure 21:**
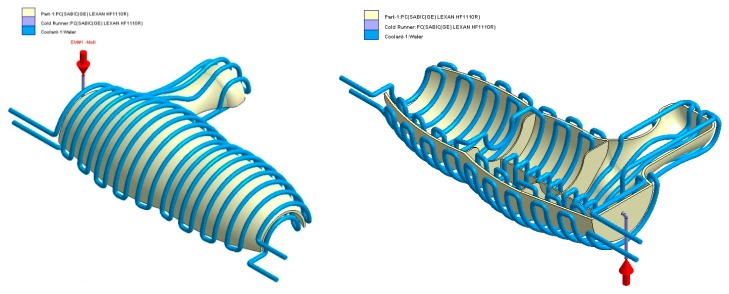
Cooling system design, Case B. Front view (**left**) and bottom view (**right**).

**Figure 22:**
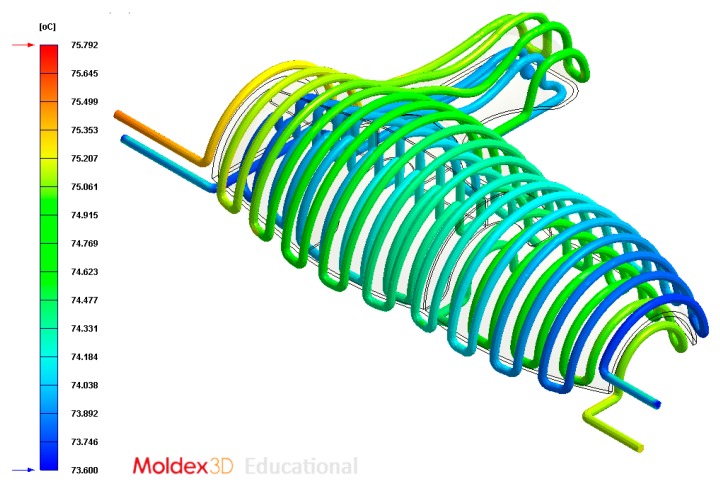
Temperature (°C) of the coolant along the conformal cooling system, Case B.

**Figure 23:**
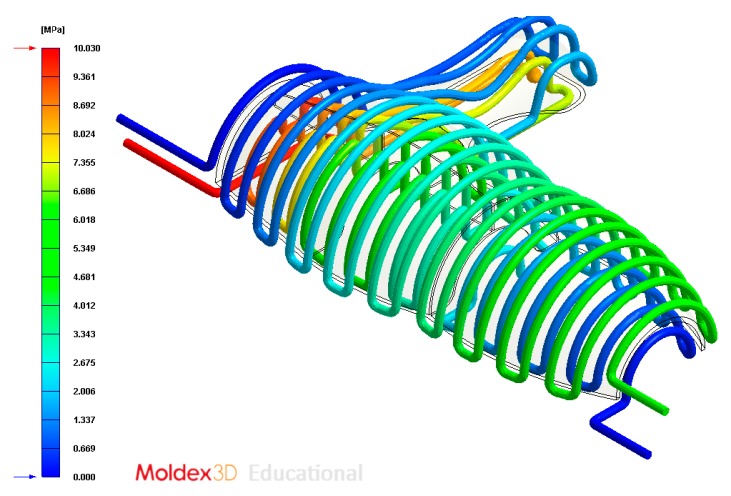
Pressure (MPa) of the coolant along the cooling system, Case B.

**Figure 24:**
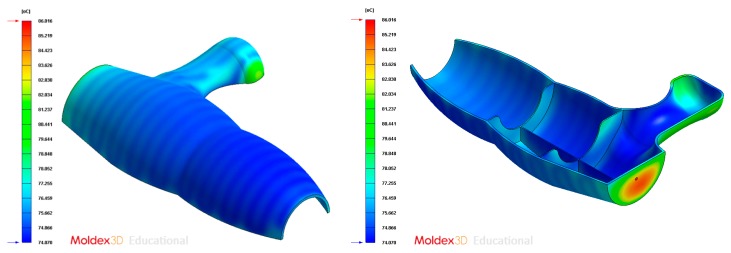
Average temperature (°C) on the surface of the plastic part after the cooling phase, Case B. Front view (**left**) and bottom view (**right**).

**Figure 25:**
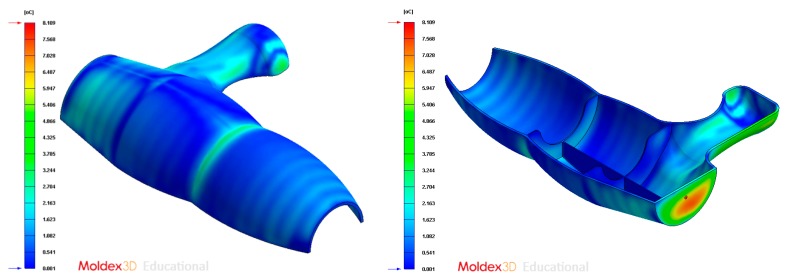
Gradient temperature (°C) on the mold surface after the cooling phase, Case B. Front view (**left**) and bottom view (**right**).

In this section the results obtained for Case B are presented in Figure 20, Figure 21, Figure 22, Figure 23, Figure 24 and Figure 25.

### 4.3. Case C

**Figure 26:**
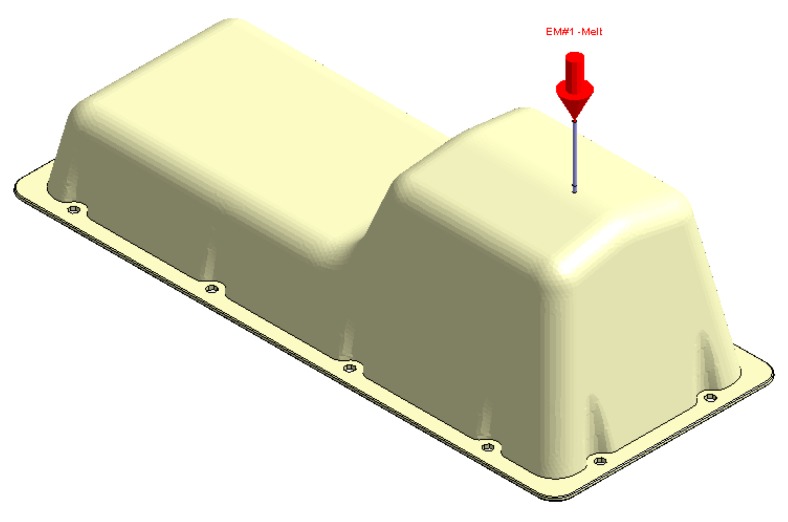
Plastic part, Case C.

**Figure 27:**
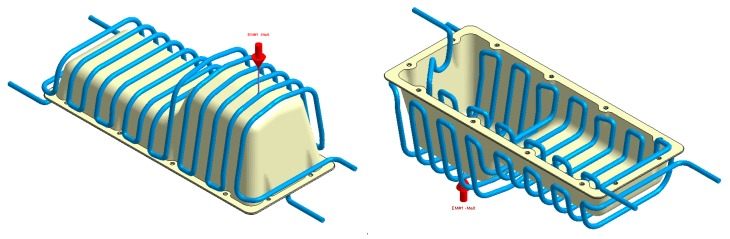
Cooling system design, Case C. Front view (**left**) and bottom view (**right**).

**Figure 28:**
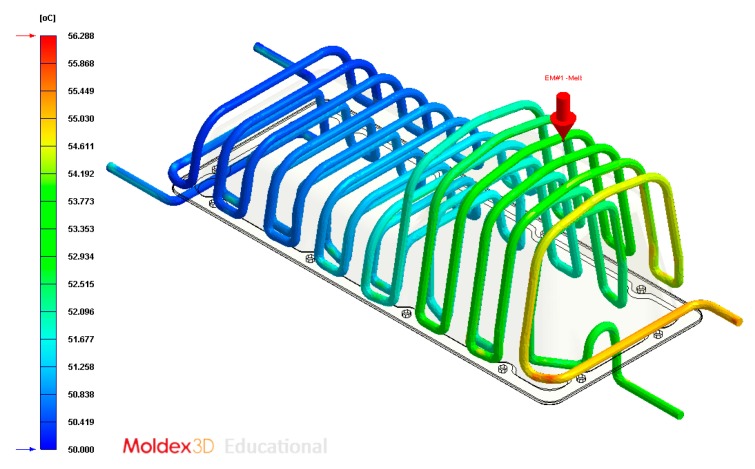
Temperature (°C) of the coolant along the conformal cooling system, Case C.

**Figure 29:**
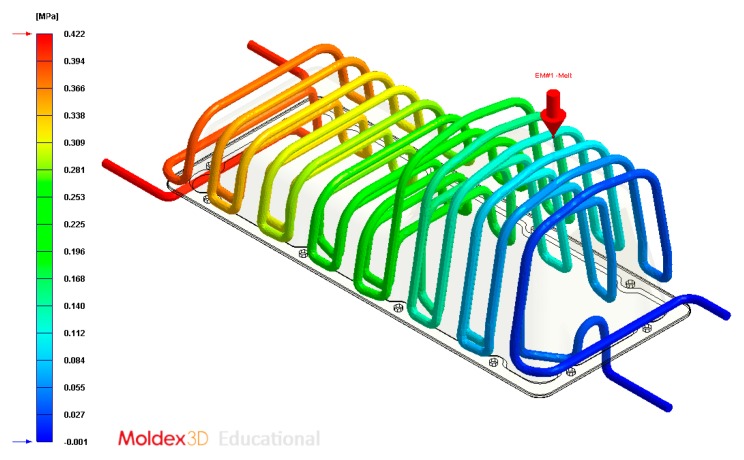
Pressure (MPa) of the coolant along the cooling system, Case C.

**Figure 30:**
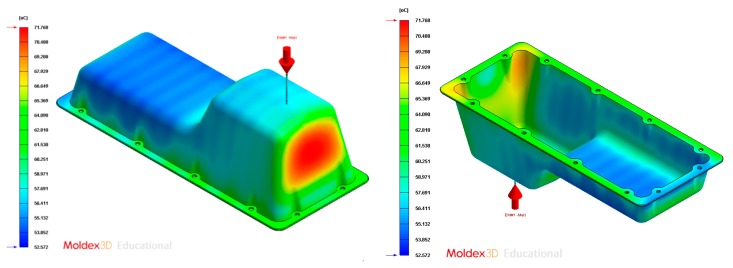
Average temperature (°C) on the surface of the plastic part after the cooling phase, Case C. Front view (**left**) and bottom view (**right**).

**Figure 31:**
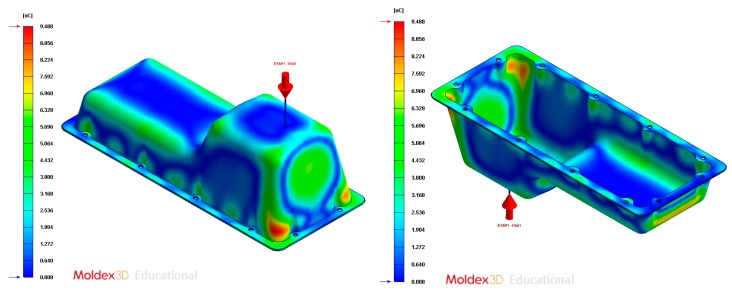
Gradient temperature (°C) on the mold surface after the cooling phase, Case C. Front view (**left**) and bottom view (**right**).

In this section the results obtained for Case C are presented in Figure 26, Figure 27, Figure 28, Figure 29, Figure 30 and Figure 31.

### 4.4. Case D

**Figure 32:**
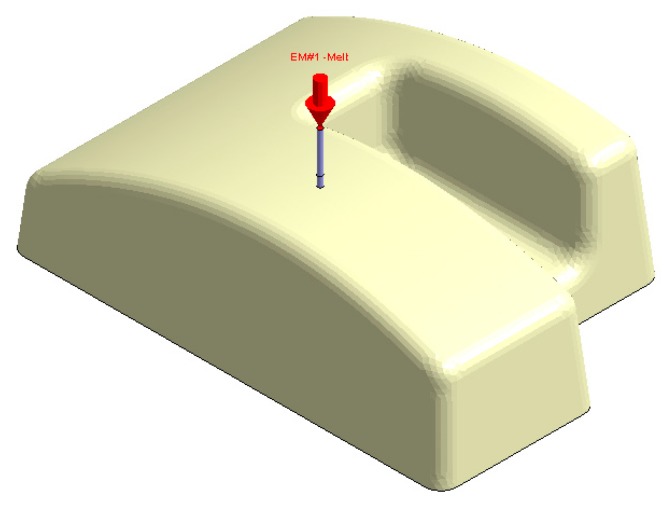
Plastic part, Case D.

**Figure 33:**
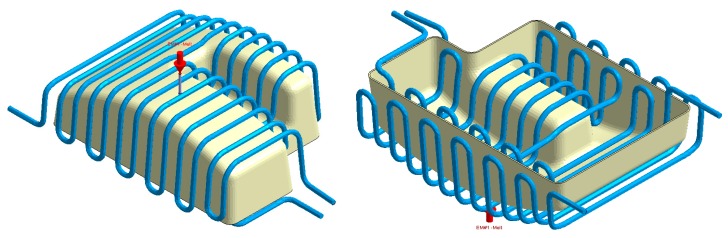
Cooling system design, Case D. Front view (**left**) and bottom view (**right**).

**Figure 34:**
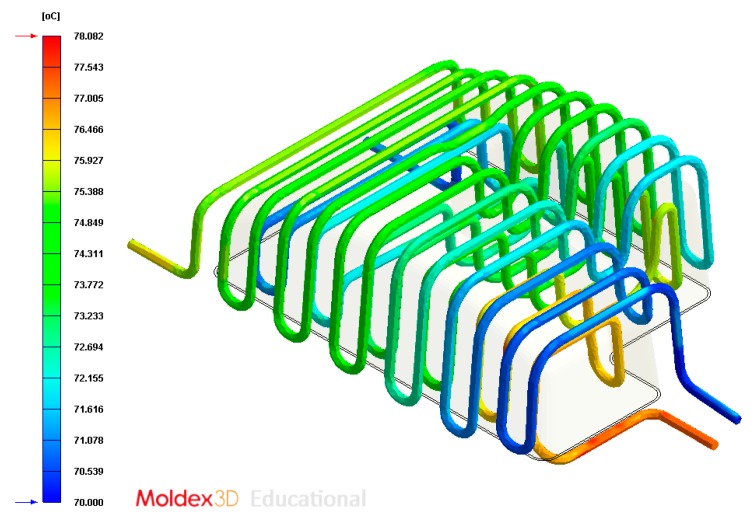
Temperatures (°C) of the coolant along the conformal cooling system, Case D.

**Figure 35:**
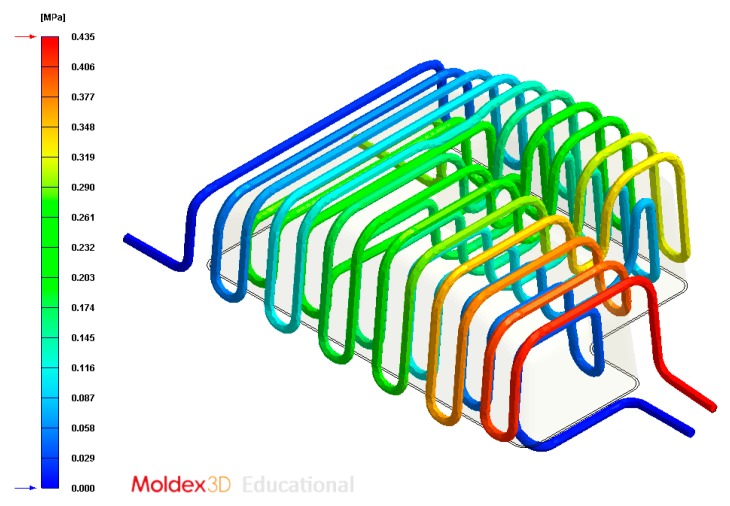
Pressure (MPa) of the coolant along the cooling system, Case D.

**Figure 36:**
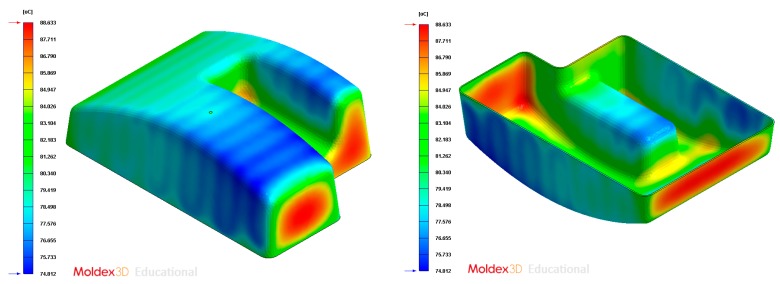
Average temperature (°C) on the surface of the plastic part after the cooling phase, Case D. Front view (**left**) and bottom view (**right**).

**Figure 37:**
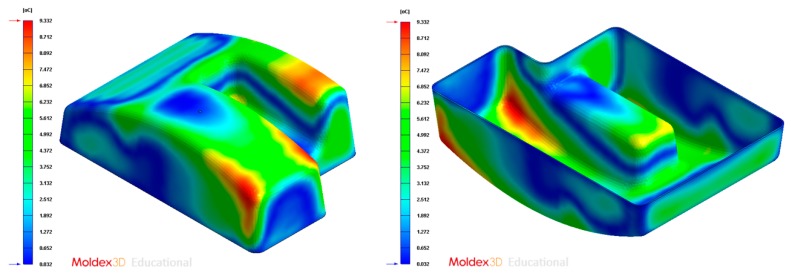
Gradient temperature field (°C) on the mold surface after the cooling phase, Case D. Front view (**left**) and bottom view (**right**).

In this section the results obtained for Case D are presented in Figure 32, Figure 33, Figure 34, Figure 35, Figure 36 and Figure 37.

## 5. Conclusions

The methodology presented in the present manuscript develops a new algorithm for the automated designing and dimensioning of the main elements of a conformal cooling system in injection molding established via the plastic part virtual mesh model and the map of temperatures of the thermoplastic filling flow during the filling phase. The algorithm groups and classifies the discrete temperature of the nodes at the end of the filling phase into geometrical areas called temperature clusters. The topological and rheological information of the clusters along with the geometrical information of the surface mesh is stored in a multidimensional discrete model of the plastic part. The methodology was evaluated by means of its implementation on four plastic parts with diverse topology and measurements. The main geometrical and technological parameters of the conformal cooling system, such as mold surface temperature, coolant inlet temperature, cooling time, distance between conformal cooling channels and plastic part, separation between conformal cooling channels, and diameters of the conformal cooling channels were dimensioned by means of expert algorithms. The layout of the conformal cooling channels for the four cases studied were obtained in an automatic way as well as detailed data reports on the results of the application. These reports included information related to conformal channel diameters D_conformal_ (mm), coordinates X, Y and Z of each conformal channel pathings (mm), the temperature of the injection mold surface T_mold_ (°C), coolant inlet temperature T_coolant_ (°C) and cooling time t_cooling_ (s).

The method presented above improves on any conventional cooling system design model since the cooling times obtained are analogous to the cooling times of analytical models including boundary conditions and ideal solutions not exceeding 5% relative error in the cases analyzed. The final quality of the plastic parts after the cooling phase meets the minimum criteria and requirements established by the injection molding companies, obtaining surface temperature differences after the cooling phase of less than 10 °C in all parts analyzed. This new methodology enhances the techniques defined until now as it does not demand heuristic methods to reach the result and does not require the feature recognition method for identifying the plastic part geometry, preventing the problems related with the CAD software and the recognition of complex features. The algorithm is appropriate for every geometry since it operates regardless of the CAD software in which the geometry was modeled. Additionally, the new algorithm applies the utility of the additive manufacturing process for rapid tooling, improving the cooling in remote zones and removing malfunction characteristics, such as warping and residual stress, thus optimizing the cycle time. The proposed algorithm admits the validation and dimensioning of the injection mold cooling system automatically and without demanding high manual computing skills and experience on the part of the injection mold designer.

## Figures and Tables

**Table 1:** Definition of the filling stage technological variables.

Nomenclature	Units	Description
t_fill_	s	Filling time of injection mold cavity
t_pack_	s	Packing time of the plastic part
t_cooling_	s	Cooling time of the plastic part
T_melt_	°C	Melt plastic flow temperature
T_mold_	°C	Injection mold temperature
T_eject_	°C	Ejection temperature of the thermoplastic material
P_inj_	MPa	Maximum filling pressure of the injection machine

**Table 2:** Optimization problem variables.

Nomenclature	Units	Description	Notation on the Optimization Problem
T_mold_	°C	Mold cavity surface temperature	x (1)
T_coolant_	°C	Coolant temperature	x (2)
L_conformal_	m	Separation distance between conformal cooling channels	x (3)
D_conformal_	m	Diameter of the conformal cooling channels	x (4)
L_p-c_	m	Separation distance between the surface of the plastic part and the conformal cooling channels	x (5) … x (11)

**Table 3:** Set-up of the genetic optimization algorithm.

Genetic Algorithm Options Structure	Defined Set-up	Description
Constraints	T_mold min_ ≤ x (1) ≤ T_mold max_ T_mold min_ ≤ x (2) ≤ T_mold max_ L_min_ ≤ x (3) ≤ L_max_ D_max_≤ x (4) ≤ D_max_ L_min_ ≤ x (5) … x (11) ≤ L_max_	Constraints restrict the value of the optimization problem variables, allowing the solution to converge on the defined domain.
Reproduction	Two Points	Future generations are created from the arithmetic mean of the two random pairs of data of the previous generation population.
Mutation	2%	Definition of the number of individuals of a generation that can be mutated in future generations.
Crossover factor	80%	Definition of the number of individuals that can be crossed in future generations.
Migration	Direction: Forward Migration Interval: 20 Migration factor: 30%	Defines the migration direction of individuals between generations and the individuals fraction that can be migrated between the computational domains.

**Table 4:** Constraint values of the optimization problem variables.

Thermoplastic Material	T_mold min_ (°C)		T_mold max_ (°C)	
Lexan: HF 1110R (PC)	80.0		120.0	
Finalloy: EBP-830 (PP)	15.0		75.0	
Cycolac: MG47 (ABS)	49.0		71.0	
**Metallic Material**	**L_min_ (mm)**	**L_max_ (mm)**	**D_min_(mm)**	**D_max_ (mm)**
Steel alloy 1.2709	3.0	20.0	3.0	6.0

**Table 5:** Magnitude of the main properties of the materials used in the numerical simulations.

Nomenclature	Units	Description	Water (Pure)	Cycolac MG47 (ABS)	Finalloy EBP-830 (PP)	Lexan HF1110R (PC)	Steel Alloy 1.2709
ρ_p_	kg/m^3^	Density	988.00	930.55	964.54	1060.40	8000
C_p_	J/kg·°C	Specific heat	4180	2314	2350	2000	450.000
δ_p_, δ_s_	W/m·°C	Thermal conductivity coefficient	0.643	0.184	0.300	0.260	20.000

**Table 6:** Definition of the filling and cooling stage technological and geometrical variables.

Nomenclature	Units	Description	Case A-Cycolac MG47 (ABS)	Case B-Lexan HF1110R (PC)	Case C-Finalloy EBP-830 (PP)	Case D-Lexan HF1110R (PC)
t_fill_	s	Filling time	1.37	1.17	2.41	2.04
t_pack_	s	Packing time	7.02	4.25	6.53	5.49
t_cooling_	s	Cooling time	30	30	30	30
T_melt_	°C	Melt temperature	238	285	235	285
T_mold_	°C	Mold temperature	64.0	91.0	69.5	84.5
T_eject_	°C	Ejection temperature	81	143	110	143
T_coolant_	°C	Coolant temperature	59.3	73.6	50.0	70.0
P_inj_	MPa	Maximum injection pressure	500	500	500	500
L_conformal_	mm	Distance between conformal cooling channels	6.00	6.25	14.40	16.20
D_conformal_	mm	Cooling channels diameter	3.9	3.0	5.3	5.9
L_p-c_	mm	Distance between plastic part and cooling channels	3.75	4.50	8.35	8.90

**Table 7:** Statistics of meshes defined for each plastic part analyzed numerically.

Description	Units	Case A-Cycolac MG47 (ABS)	Case B-Lexan HF1110R (PC)	Case C-Finalloy EBP-830 (PP)	Case D-Lexan HF1110R (PC)
Part mesh node count	-	158,775	369,904	273,488	164,946
Part mesh element count	-	382,513	885,889	593,834	352,158
Part mesh volume	m^3^	53.560 × 10^−6^	33.260 × 10^−6^	146.610 × 10^−6^	152.840 × 10^−6^
Runner mesh node count	-	21,449	33,818	15,004	23,618
Runner mesh element count	-	27,840	44,000	24,400	30,720
Runner mesh volume	m^3^	0.040 × 10^−6^	0.100 × 10^−6^	0.060 × 10^−6^	0.210 × 10^−6^
Plastic part precision (ε)–Mesh sizing	mm	1.5	1.0	2.0	1.5
Element type		Tetrahedral (10 Nodes)	Tetrahedral (10 Nodes)	Tetrahedral (10 Nodes)	Tetrahedral (10 Nodes)
Boundary layers		Prims (15 Nodes)	Prims (15 Nodes)	Prims (15 Nodes)	Prims (15 Nodes)
Offset ratio–Boundary layers		0.1	0.1	0.1	0.1

**Table 8:** Geometry properties of the plastic parts.

Geometry Properties	Units	Case A	Case B	Case C	Case D
Thickness	mm	2.5	1.5	2.0	1.5
Volume	mm^3^	53,360	33,260	146,620	152,840
Mold dimension	mm	200 × 175 × 90	240 × 180 × 70	375.800 × 200 × 190	365.200 × 280 × 170
Mold volume	mm^3^	3,048,100	2,958,200	14,007,200	17,037,100

**Table 9:** Numerical results obtained for Case A cooling analysis.

Description	Units	Value
Maximum gradient of coolant temperatures	°C	2.737
Maximum gradient of coolant pressures	MPa	3.003
Maximum temperature of the part after the cooling phase	°C	64.839
Minimum part temperature after cooling phase	°C	59.855
Maximum temperature gradient on the mold surface after the cooling phase	°C	4.590
Cooling time, analytical solution	s	11.900
Numerical cooling time, numerical solution	s	11.529
Relative error in the numerical calculation of the cooling time	%	3.118

**Table 10:** Numerical results obtained for Case B cooling analysis.

Description	Units	Value
Maximum gradient of coolant temperatures	°C	2.192
Maximum gradient of coolant pressures	MPa	10.030
Maximum temperature of the part after the cooling phase	°C	86.016
Minimum part temperature after cooling phase	°C	74.070
Maximum temperature gradient on the mold surface after the cooling phase	°C	8.109
Cooling time, analytical solution	s	5.100
Numerical cooling time, numerical solution	s	5.355
Relative error in the numerical calculation of the cooling time	%	5.000

**Table 11:** Numerical results obtained for Case C cooling analysis.

Description	Units	Value
Maximum gradient of coolant temperatures	°C	6.288
Maximum gradient of coolant pressures	MPa	0.422
Maximum temperature of the part after the cooling phase	°C	71.768
Minimum part temperature after cooling phase	°C	52.572
Maximum temperature gradient on the mold surface after the cooling phase	°C	9.488
Cooling time, analytical solution	s	5.100
Numerical cooling time, numerical solution	s	5.226
Relative error in the numerical calculation of the cooling time	%	3.255

**Table 12:** Numerical results obtained for Case D cooling analysis.

Description	Units	Value
Maximum gradient of coolant temperatures	°C	8.082
Maximum gradient of coolant pressures	MPa	0.435
Maximum temperature of the part after the cooling phase	°C	88.633
Minimum part temperature after cooling phase	°C	74.812
Maximum temperature gradient on the mold surface after the cooling phase	°C	9.332
Cooling time, analytical solution	s	4.900
Numerical cooling time, numerical solution	s	5.085
Relative error in the numerical calculation of the cooling time	%	3.776

**Figure A1:**
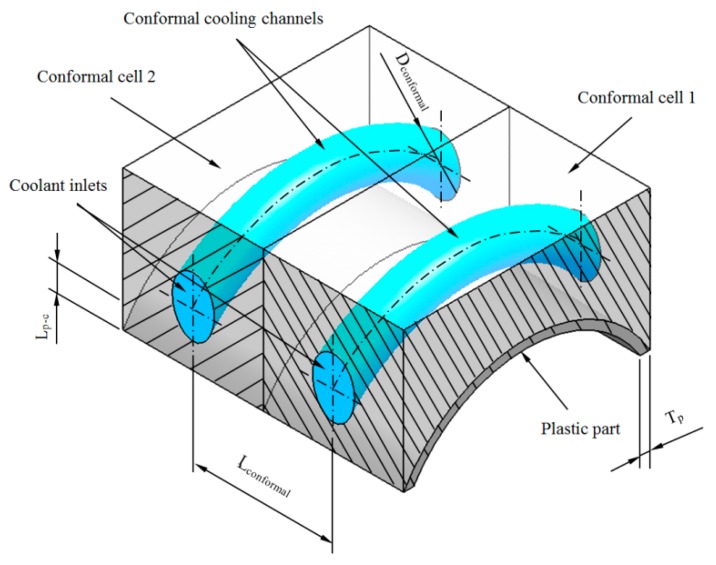
Cooling cells considered for the dimensioning of the cooling system variables.

**Figure A2:**
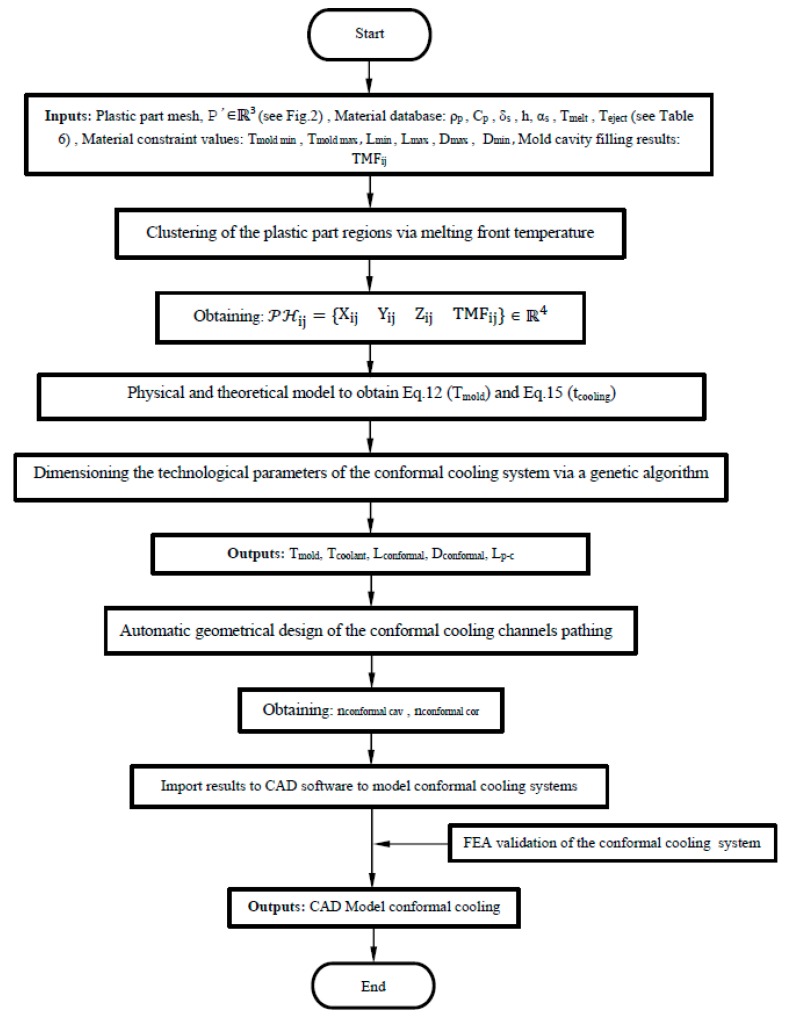
Algorithm for the design and dimensioning of the injection mold conformal cooling system.

## Appendix A

### Appendix A.1. Theoretical Model for the Study of the Conformal Cooling Channel

The theoretical model for the analytical study of the thermal exchange between the plastic part and the coolant flow which runs across the conformal cooling channels is centered on the analysis of the cooling unity. As shown in Figure A1, the cooling cell consists of a rectangular prism that contains the cross-section of the conformal cooling channel as well as the geometrical area of the corresponding plastic part. Each cooling cell is geometrically parameterized according to these variables: diameter of the conformal cooling channels D_conformal_ (m), separation between conformal cooling channels L_conformal_ (m) and the separation between the plastic part and the conformal cooling channels L_p-c_ (m) (see Figure A1 and Table A1). On the other hand, the technological variables that determine the thermal performance of the coolant flow along the conformal cooling channels are: the surface temperature of the mold cavity T_mold_ (°C) and the coolant flow temperature in the conformal cooling channels T_coolant_ (°C). The present theoretical model defines the main criteria for the automated calculation of the technological and geometrical variables of the conformal cooling channels.

**Table A1:** Geometrical and technological variables for the conformal cooling channels design.

Nomenclature	Units	Description
D_conformal_	m	Diameter of the conformal cooling channels
L_conformal_	m	Separation distance between conformal cooling channels
L_p-c_	m	Separation distance between the surface of the plastic part and the conformal cooling channels
T_mold_	°C	Injection mold temperature
T_coolant_	°C	Coolant flow temperature

Furthermore, in the present theoretical model a set of premises are defined to simplify the mathematical model, Park et al. [51].

- The mechanical and thermal characteristics of the injection mold metallic material where the conformal cooling channels are located are constant and isotropic.
- The thermal exchange of the thermoplastic filling flow is treated as unidirectional because the mold thickness is lesser than the dimensions related with XY plane.
- The thermal exchange by convection and radiation between the main elements of the injection mold and the external conditions is discounted because it corresponds to less than 5% of the total heat flow exchanged. That is to say, the thermal analysis between the plastic part and the coolant flow focuses on the thermal exchange by conduction.
- It is assumed that the coolant flow develops in a turbulent manner (Re > 4000) along the conformal cooling channels

According to Xu et al. [28], the steady temperature of the cavity mold surface, defined as T_mold_ (°C), can be formulated as Equation (A1). The set of geometric and technological variables used in Equation (A1) is shown in Table A1 and Table A2.

(A1) $$T_{\mathrm{mold}}\;=T_{\mathrm{coolant}}\;+\frac{\rho_{p}\cdot C_{p}\cdot T_{p}\cdot (2\cdot \delta_{s}\cdot L_{\mathrm{conformal}}\;+\;h\cdot \pi \cdot D_{\mathrm{conformal}}\cdot L_{p-c})\cdot (T_{\mathrm{melt}}-T_{\mathrm{eject}})}{2\cdot h\cdot \pi \cdot D_{\mathrm{cooling}}\cdot \delta s\cdot t_{\mathrm{cooling}}}$$

**Table A2:** Technological variables for the thermal exchange between the thermoplastic material and the injection mold.

Nomenclature	Units	Description
ρ_p_	kg/m^3^	Density of melt thermoplastic flow
C_p_	J/kg·°C	Specific heat of melt thermoplastic flow
δ_s_	W/m·°C	Thermal conductivity of the injection mold material
h	W/m^2^·°C	Coefficient of heat transmission between the mold and the coolant flow
α_s_	m^2^/s	Coefficient of thermal diffusivity of the injection mold material
T_melt_	°C	Temperature of the melt plastic flow
T_eject_	°C	Temperature at the plastic part ejection phase

Equation (A1) defines the temperature of the mold cavity surface. Nevertheless, the cooling time of the manufacturing process, t_cooling_, is one of the technological input variables for this equation. To establish this technological variable, the one-dimensional heat flow or Fourier equation (see Equation (A2)) is used and reduced to one dimension, in order to simplify the analytical model. According to Menges et al. [52].

(A2) $$\frac{\partial T}{\partial t}=\alpha_{s}\cdot \frac{\partial^{2}T}{\partial^{2}z}$$

where α_s_ (m^2^/s) corresponds to the coefficient of thermal diffusivity of the injection mold material. Supposing the temperature of the thermoplastic filling flow remains constant after the filling phase and equal to T_melt_ (°C) and the mold surface temperature varies until reaching a steady and constant value equivalent to T_mold_ (°C), it is plausible to define a specific solution (see Equation (A3)) for Equation (A2). The thermoplastic filling flow temperature for a point on the mold cavity surface can be expressed as a convergence of the Fourier series of Equation (A2).

(A3) $$T_{z=0}\left(t\right)=T_{\mathrm{coolant}}+\left(T_{\mathrm{melt}}-T_{\mathrm{coolant}}\right)\cdot \overset{\infty }{\underset{m=0}{\sum }}\frac{{\left(-1\right)}^{m}}{2\cdot m+1}\cdot e^{\frac{\pi \cdot {\left(2\cdot m+1\right)}^{2}\cdot \alpha_{s}}{T_{p}^{2}}\cdot t}$$

By resolving Equation (A3) regarding the cooling time variable, the equation that defines the cooling time t_cooling_ Equation (A4) is established.

(A4) $$t_{\mathrm{cooling}}=\frac{T_{p}^{2}}{\pi^{2}\cdot \alpha_{s}}\cdot \mathrm{Ln}\left(\frac{4}{\pi }\cdot \frac{T_{\mathrm{melt}}-T_{\mathrm{mold}}}{T_{\mathrm{eject}}-T_{\mathrm{mold}}}\right)$$

In this way, according to Equations (A1) and (A4), it is known that the equation that establishes the cooling time of the manufacturing process is related to the temperature of the mold cavity surface and vice versa. Therefore, the paper proposes genetic optimization algorithms in order to determine an optimal solution to the physical model as well as to measure the geometrical and technological variables (see Table A1) of the conformal cooling system. It should be noted that the methodology for dimensioning the geometrical and technological variables of the conformal cooling system is applied according to the geometrical discretization or clusters of the plastic part, based on the temperature of the melt plastic flow TMF_ij_ ∈ ℝ for each node P_ij_ ∈ P’_n_, i.e., since each geometrical discretization or cluster C_k_ (see Figure 7) is associated with an average temperature of the thermoplastic filling flow T_k_, T_k_ is used as an input to the physical model for dimensioning the conformal cooling system. In this way, the physical model presented is applied to each geometrical discretization or cluster C_k_ in order to obtain the magnitude of the geometrical and technological variables of the conformal cooling system associated with them.
